# Mitigating Contact‐Constricted Thermal Bottlenecks by Magnetically Programmable hBN Bridge Fillers in Al_2_O_3_/Epoxy Composites

**DOI:** 10.1002/smll.75261

**Published:** 2026-08-18

**Authors:** Yeonwook Jeong, Sabina Ghorsaine, Chan‐Jae Lee, In Kim, Mooho Lee, Hyejeong Lee, Ginam Kim, Jong‐Woong Kim

**Affiliations:** ^1^ Department of Semiconductor Convergence Engineering Sungkyunkwan University Suwon South Korea; ^2^ Display Research Center Korea Electronics Technology Institute Seongnam South Korea; ^3^ Samsung Electronics Device Solutions Samsung Advanced Institute of Technology Suwon South Korea

**Keywords:** advanced electronic packaging, bridge fillers, electrically insulating composites, hexagonal boron nitride, magnetic alignment, through‐plane thermal conductivity

## Abstract

Thermally conductive polymer composites often suffer from limited heat transport due to contact constriction in spherical particulate networks. To address this challenge, we report a magnetically programmable bridge‐filler architecture that enhances thermal pathways in electrically insulating epoxy systems. In this design, spherical Al_2_O_3_ serves as a process‐friendly primary filler framework, while Fe_3_O_4_‐functionalized hexagonal boron nitride (hBN@Fe_3_O_4_) flakes act as magnetically addressable bridge fillers that connect neighboring alumina particles and can be oriented along the through‐plane direction under an external magnetic field. At a fixed total filler loading of 40 vol%, the Al_2_O_3_:hBN@Fe_3_O_4_ ratio of 8:2 provides the most effective balance between bridge‐network formation and rheological freedom for alignment, yielding a through‐plane thermal conductivity of 2.02 W m^−^^1^ K^−^^1^, 72.8% higher than that of the corresponding Al_2_O_3_‐only composite. Finite‐element modeling reveals that the hybrid aligned architecture alleviates point‐contact‐dominated thermal bottlenecks by redistributing heat flux through more continuous bridge‐mediated pathways. The optimized composite retains high volume resistivity (4.4 × 10^1^ ^5^ Ω cm) and lower dielectric constant and loss than the Al_2_O_3_ composite, while package‐level thermal tests confirm faster heat transfer. These results establish a generalizable design principle for directionally efficient thermal management in electrically insulating composites with relevance to advanced electronic packaging.

## Graphical abstract

Magnetically programmable hBN bridge fillers integrated into sphere‐dominant Al_2_O_3_/epoxy composites alleviate contact‐constricted thermal bottlenecks, enabling enhanced through‐plane heat transport, electrical insulation, and directionally efficient thermal management for advanced electronic packaging.

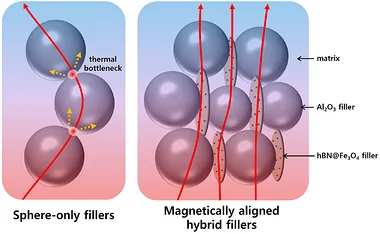

## 1 Introduction

The continued evolution of electronic systems toward higher power densities, reduced form factors, and increasingly complex packaging architectures has made thermal management a central challenge in materials design. In advanced semiconductor packaging, underfill materials are no longer required merely to fill the gap between chip and substrate or to relieve thermomechanical stress; they must also actively participate in heat dissipation while preserving electrical insulation, rheological processability, and long‐term operational reliability [1, 2, 3, 4]. These requirements become particularly stringent in densely integrated packages, where heat is generated in highly confined volumes and must be efficiently extracted across a thin vertical pathway, making underfill materials with enhanced through‐plane thermal transport an urgent priority for next‐generation packaging technologies [5, 6, 7].

Epoxy‐based polymers are among the most widely used matrix materials for underfills, encapsulants, and thermal interface structures because of their excellent dielectric characteristics, chemical resistance, and compatibility with scalable manufacturing processes [8, 9]. Yet, like most polymers, they suffer from intrinsically low thermal conductivity, typically below 0.5 W m^−^^1^ K^−^^1^ [10, 11], because heat is transported mainly through inefficient phonon transfer along structurally disordered molecular chains [12, 13, 14]. Accordingly, recent studies have explored diverse polymeric thermal management materials, including molecularly engineered epoxy composites, silicone gel composites, and advanced silicone encapsulation systems, to improve thermal and dielectric performance [15, 16, 17, 18]. Nevertheless, epoxy remains the material of choice for underfill applications, motivating extensive efforts to overcome its intrinsically low thermal conductivity by incorporating thermally conductive but electrically insulating ceramic fillers [19, 20, 21]. Among the available candidates, spherical Al_2_O_3_ has been particularly attractive for industrial underfill systems because it combines electrical insulation, moderate intrinsic thermal conductivity, relatively low cost, and favorable flow behavior at high loading. Its isotropic geometry is especially advantageous for dispensing and capillary underfill processes, where excessive viscosity or poor flowability can directly compromise manufacturability [22, 23, 24].

However, the thermal gains achievable with sphere‐only filler systems are often far more limited than expected from the intrinsic conductivity of the filler itself. In composites containing predominantly spherical particles, the filler network is built mainly through point‐to‐point contacts [25, 26]. Such contacts generate highly constricted heat‐transfer junctions with significant interfacial thermal resistance [26, 27], causing local heat‐flux concentration and inefficient phonon transmission across adjacent fillers [28]. Consequently, even when the filler loading is increased, thermal transport often saturates because the network remains geometrically discontinuous [29, 30]. From this perspective, a major bottleneck in thermally conductive underfills is not simply insufficient filler loading, but the inability of conventional sphere‐based structures to form continuous and directionally efficient heat pathways [28, 30].

Hexagonal boron nitride (hBN) is an attractive alternative filler for overcoming this limitation because it combines high intrinsic thermal conductivity with excellent electrical insulation and chemical stability [31, 32]. In particular, its 2D platelet geometry and high aspect ratio enable broader surface contacts than those produced by spherical particles, which is beneficial for reducing junction resistance and facilitating phonon transport across interconnected filler domains [33, 34, 35, 36]. Nevertheless, the direct use of large amounts of hBN flakes in epoxy is not straightforward. High‐aspect‐ratio platelets tend to cause severe viscosity increases, agglomeration, and processing difficulties, especially in formulations that must remain compatible with practical underfill operations [34]. In addition, anisotropic fillers do not automatically deliver anisotropic heat transport in the desired direction. When randomly dispersed, hBN platelets may form statistically distributed conductive domains, but such domains are not necessarily organized to support efficient heat dissipation across the chip–substrate thickness. Since the dominant thermal pathway in underfill applications is through‐plane rather than in‐plane, controlling filler orientation is as important as selecting the filler itself.

These considerations suggest that the central design challenge lies in simultaneously balancing processability, electrical insulation, and thermally efficient network formation. Hybrid filler strategies have therefore emerged as a promising route because they combine fillers with different geometries and functions. In such systems, spherical fillers can provide a process‐friendly high‐loading framework, whereas anisotropic fillers can bridge neighboring particles, reduce polymer‐rich gaps, and improve inter‐filler connectivity [37, 38]. Importantly, this hybrid concept is not merely a compositional mixing strategy; it is a structural approach for engineering network topology. When appropriately designed, flake‐like bridge fillers can convert isolated point contacts into more extended and continuous conduction pathways, thereby alleviating contact‐limited heat‐transfer bottlenecks. Niu et al. [39] reported that at a total filler loading of 50 wt%, the hybrid filler system of hBN flake and spherical Al_2_O_3_ in the ratio 3:2 increased the through‐plane thermal conductivity to 2.2 W m^−^^1^ K^−^^1^, compared with 0.7 W m^−^^1^ K^−^^1^ for the Al_2_O_3_‐only composite. Pan et al. [40] also reported that, at a total filler loading of 30 vol%, the hybrid filler system of AlN particles and hBN platelets in the ratio 2:1 increased the through‐plane thermal conductivity from 0.68 W m^−^^1^ K^−^^1^ to 1.04 W m^−^^1^ K^−^^1^ for the hybrid composite. Yet in most reported hybrid composites, the fillers remain randomly distributed, which means that bridging occurs only locally and the resulting thermal network still lacks directional coherence, particularly in the through‐plane direction that is most relevant for packaging applications [41, 42].

A more effective solution would be to actively program both the connectivity and orientation of the hybrid filler network. External‐field‐assisted alignment offers such an opportunity because it allows anisotropic fillers to be organized along a target direction during composite formation. Among the available approaches, magnetic alignment is particularly attractive because it can provide directional control without imposing direct electrical contact or complex mechanical templating [43, 44]. For example, Kim and Kim fabricated Fe_3_O_4_‐coated BN nanoplatelets by liquid‐phase exfoliation and vertically aligned them in epoxy under a 0.4 T magnetic field. At 20 wt% filler loading the aligned composite reached 1.07 W m^−^^1^ K^−^^1^, corresponding to a 127.7% increase over the unaligned BNNP/Fe_3_O_4_ composite and a 72.6% increase over the raw BN composite [45]. Gurijala et al. [46] demonstrated that magnetic‐field alignment of hBN at 60 vol% in an epoxy matrix enhanced the through‐plane thermal conductivity, reaching 7.28 W m^−^^1^ K^−^^1^ compared with 2.56 W m^−^^1^ K^−^^1^ for the non‐aligned hBN composite. Other recent studies have also extended magnetic alignment to BN nanosheet/epoxy composite films and magnetic‐field‐assisted 3D printing of Fe_3_O_4_‐decorated BN composites, further confirming the effectiveness of field‐directed BN orientation for constructing anisotropic heat‐transfer pathways [47, 48]. Beyond BN‐based systems, Cheng et al. [49] also showed that coplanar alignment of graphene nanoplatelets at 72 wt% in polyvinyl alcohol under a magnetic field enhanced the through‐plane thermal conductivity, increasing it from 1.14 W m^−^^1^ K^−^^1^ to 11.78 W m^−^^1^ K^−^^1^, highlighting the broader applicability of magnetic alignment to anisotropic thermally conductive fillers. These studies demonstrate that if thermally conductive anisotropic fillers can be endowed with magnetic responsiveness, they may serve not only as passive fillers but as field‐directable structural elements that reorganize the composite network during curing. This opens a route to decouple two requirements that are often difficult to satisfy simultaneously in thermally conductive underfills: the processability associated with sphere‐dominant formulations and the directional heat‐transport efficiency enabled by aligned anisotropic bridges.

In this paper, we report a magnetically programmable bridge‐filler architecture designed to overcome the thermal bottlenecks in traditional underfill composites. By employing spherical Al_2_O_3_ as a process‐friendly framework and Fe_3_O_4_‐functionalized hBN flakes as magnetically responsive bridges, we demonstrate a strategy to reconfigure filler networks at micro‐ and nanoscale levels. Rather than relying solely on increased filler loading or on the use of intrinsically more conductive components, this strategy focuses on reconfiguring how fillers bridge, contact, and align across micro‐ and nanoscale length scales. As a result, the composite network is transformed from a contact‐limited particulate assembly into a more continuous and directionally organized heat‐transfer structure [50, 51, 52]. On the basis of this concept, we demonstrate that aligned hBN@Fe_3_O_4_ bridges substantially enhance through‐plane thermal conductivity while preserving low dielectric constant and loss tangent. Our findings, supported by experimental data and finite‐element modeling, provide a generalizable principle for engineering high‐performance thermal networks while maintaining the essential dielectric properties required for advanced electronic packaging.

## 2 Results and Discussion

### 2.1 Design and Validation of a Magnetically Programmable hBN Bridge Filler

Figure 1 presents the central design concept of this work, in which hexagonal boron nitride (hBN) flakes were transformed into magnetically programmable bridge fillers and integrated with spherical Al_2_O_3_ to construct a directionally organized thermal network in an epoxy matrix. Rather than relying solely on increasing filler loading, this strategy addresses the geometric origin of inefficient heat transport in sphere‐dominant composites. Because spherical fillers predominantly form discrete point contacts, heat flow is readily constricted at localized junctions. In contrast, anisotropic hBN flakes can bridge neighboring spherical particles and create more extended interfacial contacts that are more favorable for phonon transport. On this basis, hBN was selected as the thermally conductive bridging phase, while Fe_3_O_4_ functionalization was introduced to render these flakes magnetically addressable during composite fabrication.

**FIGURE 1:**
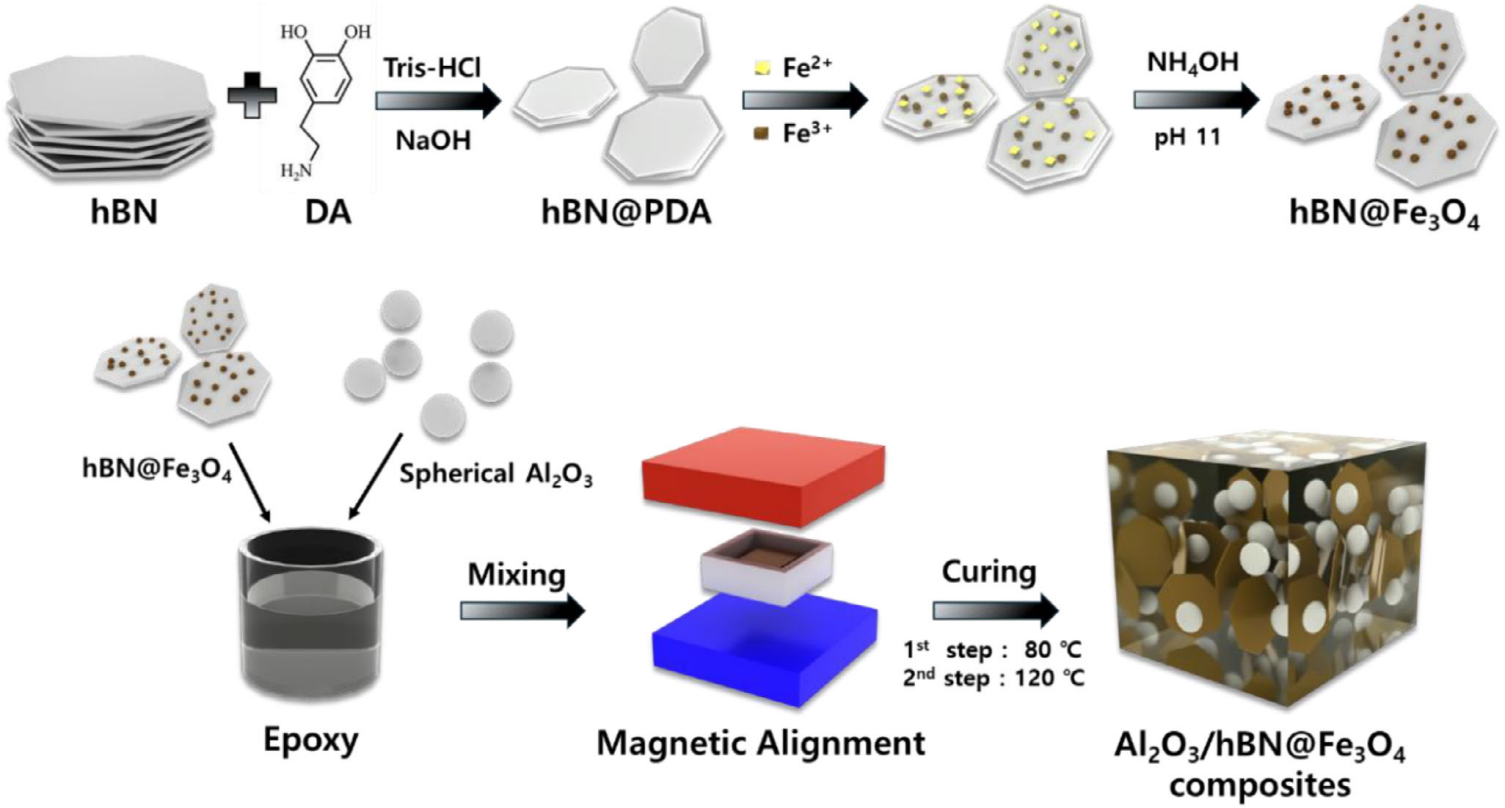
Schematic illustration of the concept and fabrication strategy of magnetically programmable hBN bridge fillers in Al_2_O_3_/epoxy composites.

The interfacial design of Fe_3_O_4_‐functionalized hexagonal boron nitride (hBN@Fe_3_O_4_) is particularly important from a structure–function standpoint. In the present system, hBN must retain its platelet geometry to operate as an effective bridge filler, whereas Fe_3_O_4_ must be introduced in a manner that confers magnetic responsiveness without disrupting this anisotropic morphology. To satisfy these requirements, hBN was first coated with polydopamine (PDA), which serves as both an adhesive and a coordination‐active interlayer between the chemically inert hBN surface and the subsequently formed Fe_3_O_4_ domains [53, 54, 55]. This PDA‐mediated route enables iron‐ion adsorption and surface‐confined Fe_3_O_4_ nucleation while preserving the bridge‐forming geometry of the parent flake. A more detailed discussion of this interfacial rationale is provided in Note S1 together with Figures S1–S4.

The successful construction of hBN@Fe_3_O_4_ was first verified morphologically. As shown in Figure 2a, the functionalized filler largely retains the characteristic platelet morphology of hBN, with a lateral size of approximately 5 µm. Comparison with the pristine fillers in Figure S1 confirms that the parent hBN geometry is preserved after surface modification, whereas high‐magnification SEM images in Figure S2a reveal that nanoscale particles with diameters of roughly 50 nm are distributed over the flake surface. This observation is important because excessive particle overgrowth or severe aggregation would compromise the bridge‐filler function by blunting the platelet geometry or disrupting interparticle spanning. In addition, the elemental maps in Figure 2b and Figure S2b show the spatial coexistence of B, N, Fe, and O throughout the flakes, indicating that Fe‐containing oxide domains were formed directly on hBN rather than being physically admixed as separate particles. Comparison with the pristine fillers and the additional high‐magnification and spectroscopic data in Figures S1–S3 further supports that magnetic functionalization proceeded through surface decoration rather than structural disruption of the hBN flakes (seeNote S1).

**FIGURE 2:**
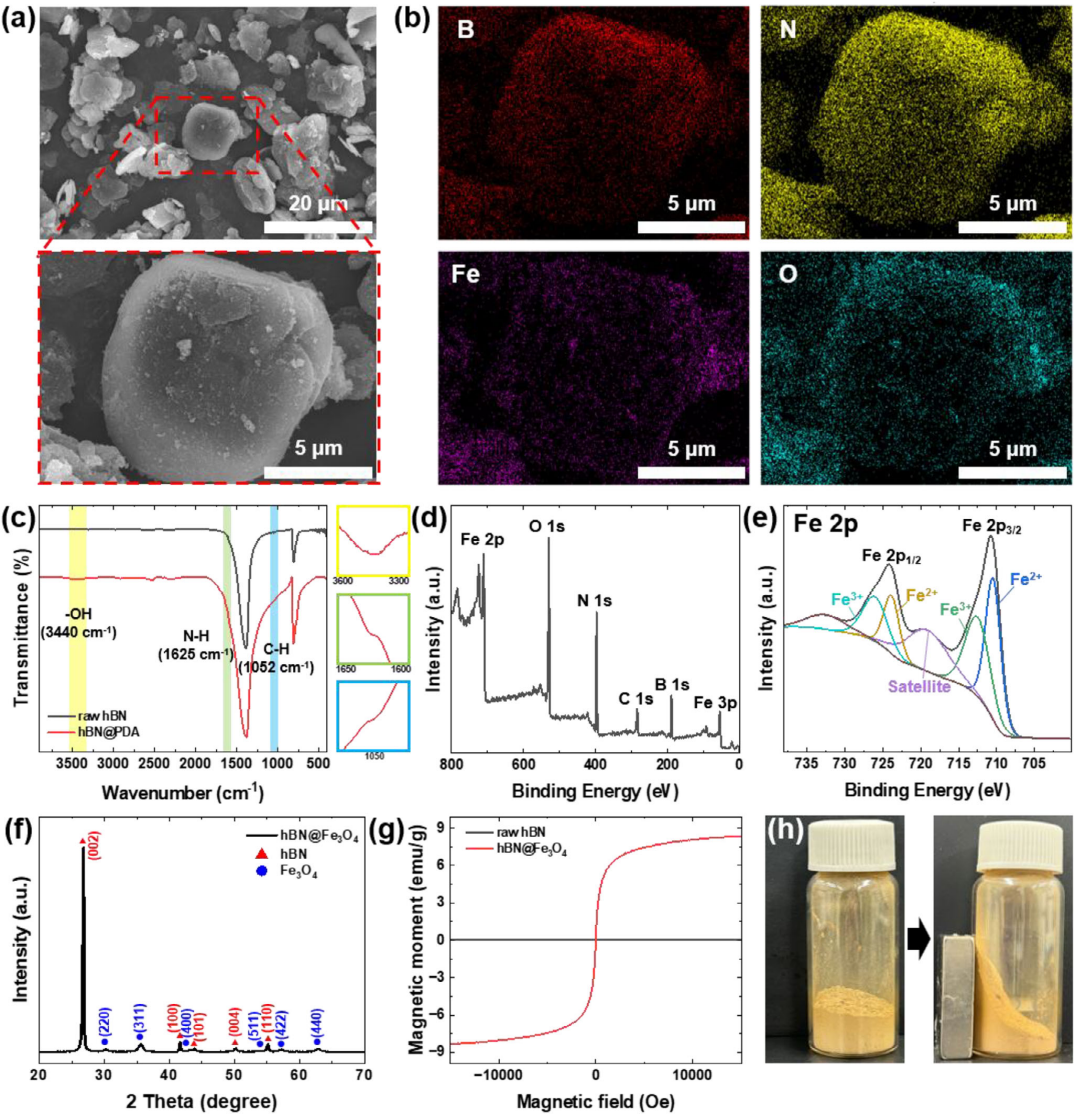
Structural, chemical, and magnetic validation of hBN@Fe_3_O_4_ bridge fillers. (a) FE‐SEM image of hBN@Fe_3_O_4_, showing retention of the platelet morphology after magnetic functionalization. (b) EDS elemental mapping images of B, N, Fe, and O, confirming the spatial coexistence of the hBN framework and Fe‐containing surface domains. (c) FT‐IR spectra of pristine hBN and PDA‐coated hBN, showing the formation of the PDA interfacial layer. (d) XPS survey spectrum of hBN@Fe_3_O_4_. (e) High‐resolution Fe 2p XPS spectrum of hBN@Fe_3_O_4_, confirming the presence of mixed‐valence iron species characteristic of magnetite. (f) XRD patterns of pristine hBN and hBN@Fe_3_O_4_, showing preservation of the hBN crystalline framework together with additional reflections attributable to Fe_3_O_4_. (g) Magnetic hysteresis loops of pristine hBN and hBN@Fe_3_O_4_ measured at room temperature. (h) Photograph showing the magnetic response of hBN@Fe_3_O_4_ under an external magnetic field.

The sequential surface‐functionalization process was further supported by spectroscopic analysis. In the FTIR spectra (Figure 2c), pristine hBN exhibits the characteristic B–N out‐of‐plane bending and in‐plane stretching modes, whereas PDA‐coated hBN shows additional absorption features associated with PDA‐derived organic functionalities, indicating successful formation of the interfacial coating layer. This interlayer is structurally important because it couples the chemically inert hBN surface with the subsequent inorganic deposition step. The XPS survey spectrum of hBN@Fe_3_O_4_ (Figure 2d) displays B 1s, N 1s, O 1s, C 1s, and Fe 2p signals simultaneously, while the high‐resolution Fe 2p spectrum (Figure 2e) reveals mixed‐valence iron states characteristic of magnetite [56, 57]. To further clarify the surface chemistry, the high‐resolution B 1s, N 1s, O 1s, and C 1s spectra are provided in Figure S3. In particular, the C 1s and N 1s components support the presence of PDA‐derived organic species on the hBN surface, whereas the oxygen‐containing components are consistent with the coexistence of PDA‐associated functionalities and iron oxide species [58]. Together, these FTIR and XPS results indicate that Fe_3_O_4_ was not simply introduced as a physically blended additive, but was chemically integrated onto a PDA‐functionalized hBN interface.

The structural integrity of the filler after magnetic functionalization was additionally confirmed by XRD (Figure 2f) [59]. The characteristic diffraction peaks of hBN remain clearly visible after surface treatment, while additional reflections attributable to Fe_3_O_4_ appear simultaneously. This result suggests that the crystalline framework of the parent hBN flakes is preserved while a magnetite phase is successfully introduced onto the surface. Preserving this underlying hBN structure is important not only compositionally but also functionally, because the bridge‐filler concept depends on maintaining the intrinsic thermal‐networking capability of the anisotropic boron nitride phase.

Most importantly, the resulting hBN@Fe_3_O_4_ flakes exhibit clear magnetic responsiveness. As shown in Figure 2g, pristine hBN displays negligible magnetization, whereas hBN@Fe_3_O_4_ shows a distinct magnetic hysteresis response with a saturation magnetization of approximately 8.3 emu g^−^^1^ and only small coercivity and remanence [59]. The visible attraction of the powder toward an external magnet in Figure 2h further confirms that the functionalized flakes can respond under practically accessible magnetic‐field conditions. For the present materials design, this point is crucial: the role of Fe_3_O_4_ is not to serve as the principal thermally conductive phase, but to provide a magnetic handle that enables active reorganization of thermally conductive hBN bridges during composite formation. Once this functionality is introduced, the filler system is no longer limited to stochastic packing, but can in principle be guided toward a directionally coherent network that is better aligned with through‐plane heat dissipation. Because the Fe_3_O_4_ loading must balance magnetic responsiveness against preservation of the intrinsic heat‐transport capability of hBN, the amount of Fe_3_O_4_ deposited on the flakes was additionally optimized. Among the investigated formulations, hBN@Fe_3_O_4_–2.5, containing approximately 11.3 wt% Fe_3_O_4_, exhibited the highest aligned through‐plane thermal conductivity and was therefore selected for the main study (see Note S2 together with Figures S5–S7).

Figures 1 and 2, together with the supporting evidence in Figures S1–S4, validate the key materials premise of this study. hBN can be transformed into a magnetically programmable bridge filler while retaining its platelet morphology and thermal‐networking potential, thereby establishing the structural foundation for directional thermal‐pathway engineering in sphere‐dominant polymer composites. This bridge‐filler platform provides the basis for the next stage of the study, where magnetic alignment within Al_2_O_3_/hBN@Fe_3_O_4_/epoxy composites is examined as a strategy to alleviate contact‐limited thermal bottlenecks in electrically insulating thermal‐management materials.

### 2.2 Rheologically Gated Magnetic Alignment of Hybrid Bridge Fillers

Having established in Section 2.1 that hBN can be converted into a magnetically programmable bridge filler, the next question is whether this functionality can be translated into directional organization inside a sphere‐dominant epoxy composite. For the present materials strategy, magnetic responsiveness alone is not sufficient; the flakes must be able to physically reorient within the uncured matrix before the network is locked by curing. Figure 3 therefore examines the magnetic‐field‐induced alignment behavior of Al_2_O_3_/hBN@Fe_3_O_4_ hybrid fillers from four complementary perspectives: macroscopic field response, rheological processability, microstructural evidence of orientation, and quantitative orientation analysis.

**FIGURE 3:**
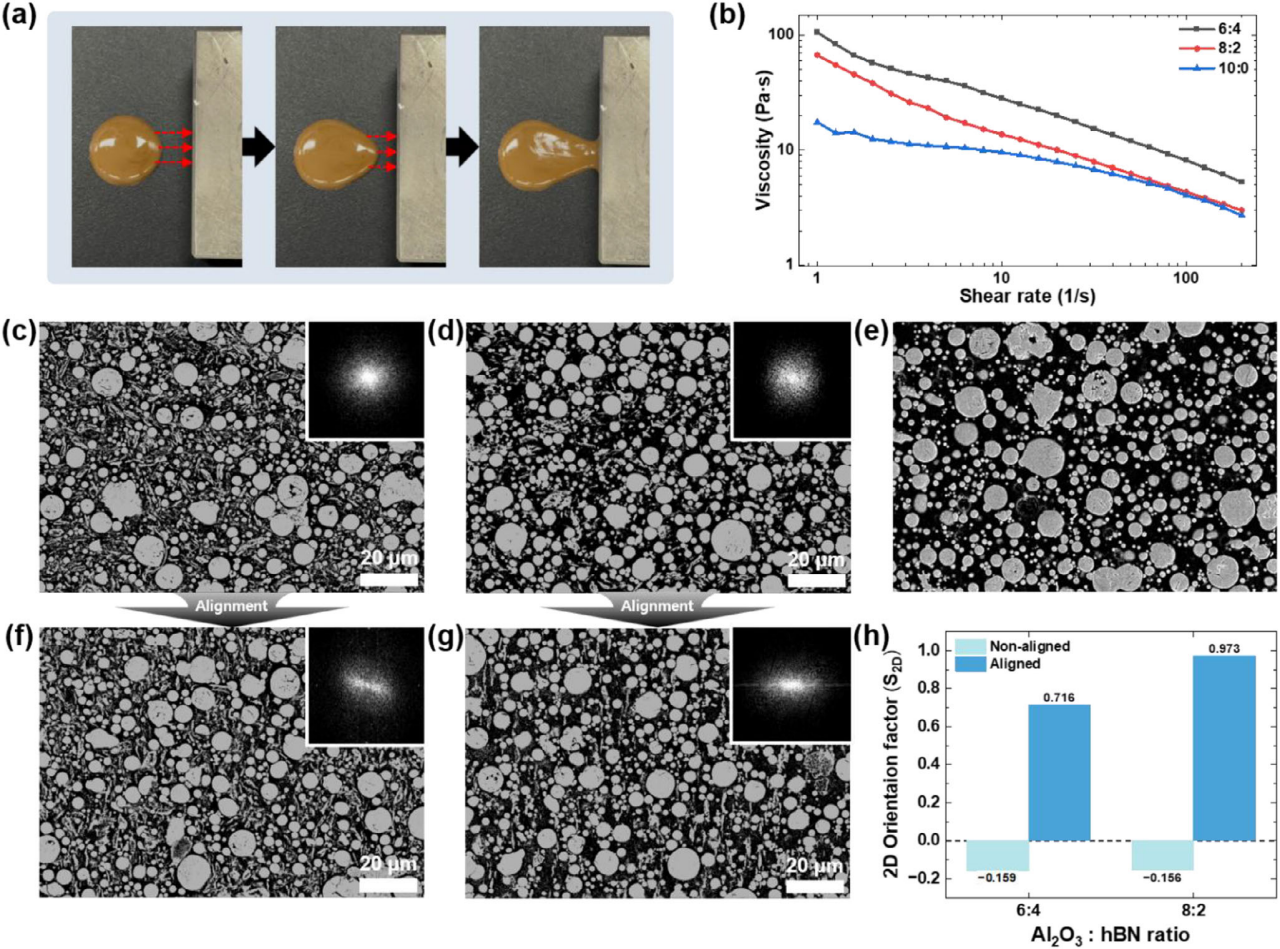
Rheologically gated magnetic alignment of Al_2_O_3_/hBN@Fe_3_O_4_ hybrid fillers in epoxy composites. (a) Photograph showing the field responsiveness of an uncured Al_2_O_3_/hBN@Fe_3_O_4_/epoxy mixture under an external magnetic field. (b) Viscosity of uncured Al_2_O_3_/hBN@Fe_3_O_4_/epoxy mixtures with Al_2_O_3_:hBN@Fe_3_O_4_ ratios of 10:0, 8:2, and 6:4 at a fixed total filler loading of 40 vol%, showing composition‐dependent rheological resistance to alignment. (c,d) Cross‐sectional SEM images and corresponding FFT patterns of non‐aligned Al_2_O_3_/hBN@Fe_3_O_4_/epoxy composites with Al_2_O_3_:hBN@Fe_3_O_4_ ratios of 6:4 and 8:2, respectively. (e) Cross‐sectional SEM image of the Al_2_O_3_‐only/epoxy composite. (f,g) Cross‐sectional SEM images and corresponding FFT patterns of magnetically aligned Al_2_O_3_/hBN@Fe_3_O_4_/epoxy composites with Al_2_O_3_:hBN@Fe_3_O_4_ ratios of 6:4 and 8:2, respectively. (h) Orientation factors of hBN@Fe_3_O_4_ fillers before and after magnetic alignment.

A simple but important qualitative indication of magnetic programmability is provided by Figure 3a. When an uncured composite containing Al_2_O_3_:hBN@Fe_3_O_4_ = 8:2 at a total filler loading of 40 vol% was placed near an external magnet, the sample exhibited visible movement toward the magnetic field. This observation indicates that the magnetic response identified for the isolated hBN@Fe_3_O_4_ flakes in Figure 2 is retained even after incorporation into the viscous epoxy/filler mixture. In other words, the bridge filler remains magnetically addressable in the composite state, which is a prerequisite for field‐directed reorganization during processing. This interpretation is further supported by Figure S8, where even an epoxy composite containing only hBN@Fe_3_O_4_ flakes shows a clear change in filler orientation after magnetic‐field exposure.

At the same time, field‐induced alignment in composite media is inherently constrained by rheology. The anisotropic hBN@Fe_3_O_4_ flakes can rotate under a magnetic field only when the magnetic torque acting on the filler exceeds the viscous resistance imposed by the surrounding matrix [60]. In a simplified description, the magnetic torque can be expressed as:

(1) $$\tau_{m}\approx \frac{\Delta \chi VB^{2}}{2\mu_{0}}\sin\left(2\theta \right)$$

where μ_0_ is the vacuum permeability, Δχ is the effective magnetic anisotropy of the filler, *V* is the effective filler volume, *B* is the magnetic‐field strength, and θ is the angle between the filler orientation and the field direction. By contrast, rotational reorientation in a viscous medium is opposed by a drag torque of the form:

(2) $$\tau_{v}\approx \zeta_{r}\eta \frac{d\theta }{dt}$$

where η is the viscosity of the suspension and ζ_*r*_ is a rotational drag coefficient associated with filler geometry. Under the overdamped conditions relevant to uncured composite slurries, alignment is therefore governed by the competition between magnetic driving and viscous dissipation, meaning that the characteristic alignment time increases with increasing viscosity for a given magnetic field and particle geometry. A more detailed discussion of this torque‐balance framework is provided in Note S3.

This rheological gating is directly reflected in Figure 3b. At a fixed total filler loading of 40 vol%, all three compositions exhibit typical shear‐thinning behavior, but the viscosity increases systematically with increasing hBN@Fe_3_O_4_ content, following the order 6:4 > 8:2 > 10:0 over the entire shear‐rate range. At a shear rate of 1 s^−^^1^, the viscosities of the 10:0, 8:2, and 6:4 composites are 17.6, 67.2, and 106.0 Pa·s, respectively; even at 200 s^−^^1^, the same trend remains, with viscosities of 2.71, 3.01, and 5.29 Pa·s. This increase is consistent with the high‐aspect‐ratio geometry of hBN@Fe_3_O_4_, which intensifies hydrodynamic resistance and filler–filler interactions compared with sphere‐only systems [61]. Thus, although increasing the fraction of anisotropic bridge fillers may appear beneficial from a connectivity standpoint, it also imposes a growing rheological penalty that can hinder their field‐driven reorientation.

The resulting alignment behavior was visualized by cross‐sectional SEM and corresponding FFT analysis of the non‐aligned and aligned hybrid composites (Figure 3c,d,f,g). Figure 3e shows the cross‐sectional morphology of the Al_2_O_3_‐only composite and provides a reference for the sphere‐dominant particulate backbone in the absence of hBN@Fe_3_O_4_ bridge fillers. Before magnetic‐field treatment, both the 8:2 and 6:4 composites show relatively disordered distributions of hBN@Fe_3_O_4_ flakes, and their FFT patterns remain comparatively isotropic, indicating the absence of a distinct preferred orientation. After field application, however, the flakes become preferentially oriented along the applied‐field direction, and the FFT patterns develop clear anisotropic features. These results demonstrate that the magnetically responsive bridge fillers are not merely dispersed in the composite, but can be actively reorganized into a directionally biased microstructure during the pre‐curing stage.

To quantitatively evaluate this reorientation, the projected orientations of individual hBN@Fe_3_O_4_ platelet traces were measured from the cross‐sectional SEM images. Because the platelets are nonpolar, orientations differing by 180° were treated as equivalent. The measured ImageJ angle, α, was referenced to the horizontal direction and converted to the projected misorientation angle relative to the through‐plane direction as $\phi =|\alpha -90^{\circ }|$. The preferential orientation was then quantified using the 2D orientational order parameter, *S*_2D_, which is the 2D analogue of the Hermans orientation factor [62]:

(3) $$S_{2D}=2\langle \mathrm{co}s^{2}\phi \rangle -1$$

With the through‐plane direction used as the fixed reference axis, *S*_2D_ =  0 represents a random projected orientation distribution, whereas *S*_2D_ =  1 and − 1 represent perfect through‐plane and in‐plane alignment, respectively. Before magnetic alignment, the 6:4 and 8:2 composites exhibited similar *S*_2D_ values of −0.159 and −0.156, respectively, indicating a weak initial in‐plane bias. Following magnetic‐field treatment, the corresponding values increased markedly to 0.716 and 0.973 (Figure 3h). The near‐unity value obtained for the 8:2 composite confirms a highly pronounced projected through‐plane orientation, whereas the lower value for the 6:4 composite indicates less complete reorientation. The corresponding orientation‐angle distributions are provided in Figure S9.

These quantitative differences confirm that the final degree of anisotropy is determined not only by magnetic responsiveness, but also by the balance between the magnetic alignment force and rheological resistance. In the 8:2 composite, the flakes exhibit clear preferential alignment after field exposure, indicating that the viscosity remains low enough to permit substantial field‐induced rotation while still maintaining a sufficient bridge‐filler fraction to reorganize local connectivity. By contrast, in the 6:4 composite, the higher flake content increases the probability of inter‐filler interactions and viscosity buildup, thereby suppressing rotational mobility and reducing the net effectiveness of alignment despite the larger population of magnetically responsive flakes. The practical implication is that alignment efficiency in hybrid composites does not monotonically increase with bridge‐filler content; instead, it is maximized within a processing window where magnetic addressability and rheological freedom are optimally balanced.

An additional point is that the global anisotropy signal of a hybrid composite may not fully capture the extent of thermal‐pathway reorganization. In sphere‐dominant systems such as the present composites, even a relatively modest fraction of aligned flake bridges can exert a disproportionately large effect on interparticle connectivity, because these bridges selectively reduce contact‐limited interruptions between neighboring Al_2_O_3_ particles. This is why microstructural alignment should be interpreted not simply in terms of visual anisotropy alone, but in terms of its topological consequence for heat‐flow continuity [63]. Supporting this interpretation, Figure S8 shows that hBN@Fe_3_O_4_ flakes are intrinsically capable of field‐induced orientation in the epoxy matrix, while the hybrid composites reveal that the extent to which this intrinsic alignability is expressed is strongly modulated by composite rheology.

Taken together, Figure 3 establishes that magnetic alignment in Al_2_O_3_/hBN@Fe_3_O_4_/epoxy composites is a rheologically gated structural process rather than a simple consequence of filler magnetization. The hBN@Fe_3_O_4_ bridge fillers remain magnetically addressable after composite incorporation, but their actual orientation efficiency depends critically on whether the uncured matrix provides sufficient rotational freedom. This finding is central to the present design strategy, because it identifies the 8:2 hybrid composition at 40 vol% total filler loading as a regime where field‐directed bridge formation is possible without overwhelming the processability advantage of the sphere‐dominant matrix. On this basis, the following section examines how such composition‐ and alignment‐dependent structural organization translates into through‐plane thermal transport performance.

### 2.3 Topology‐Optimized Through‐Plane Thermal Transport in Hybrid Composites

Having established that hBN@Fe_3_O_4_ can function as a magnetically programmable bridge filler and that its field‐induced orientation is strongly gated by composite rheology, the next step is to determine how this structural reorganization translates into heat‐transport performance. Figure 4 addresses this question at three levels: first, by identifying the optimal hybrid filler composition at a fixed total loading; second, by examining how the thermal benefit of aligned bridge fillers evolves with increasing filler content; and third, by comparing the thermal performance of the optimized composite with previously reported polymer composites employing BN/Al_2_O_3_, Al_2_O_3_‐only, and other hybrid filler systems. Rather than treating thermal conductivity as a simple function of filler fraction alone, this analysis reveals that the decisive parameter is the extent to which bridge fillers can reorganize heat‐flow topology under processing‐accessible conditions.

**FIGURE 4:**
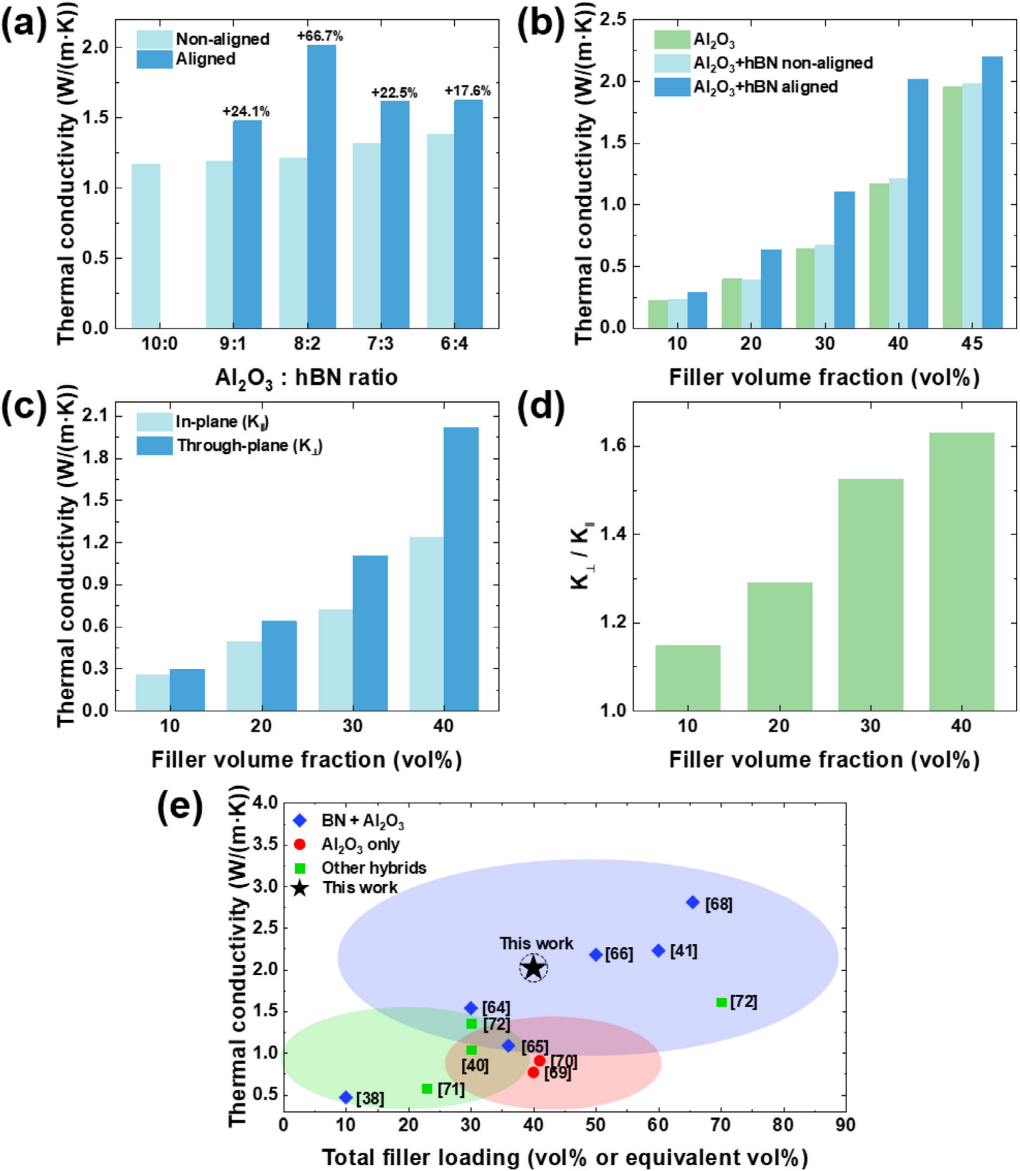
Composition‐ and loading‐dependent optimization of through‐plane thermal transport in hybrid aligned composites. (a) Through‐plane thermal conductivity of Al_2_O_3_/hBN@Fe_3_O_4_/epoxy composites as a function of Al_2_O_3_:hBN@Fe_3_O_4_ ratio (10:0, 9:1, 8:2, 7:3, and 6:4) at a fixed total filler loading of 40 vol%, before and after magnetic‐field‐induced alignment, showing an optimum at 8:2. (b) Through‐plane thermal conductivity of Al_2_O_3_‐only and Al_2_O_3_:hBN@Fe_3_O_4_ (8:2) composites as a function of total filler loading (10, 20, 30, 40, and 45 vol%). (c) Comparison of through‐plane and in‐plane thermal conductivity of aligned Al_2_O_3_:hBN@Fe_3_O_4_ (8:2) composites as a function of total filler loading. (d) Thermal‐conductivity anisotropy ratio of aligned Al_2_O_3_:hBN@Fe_3_O_4_ (8:2) composites as a function of total filler loading. The figure shows that the highest through‐plane conductivity is achieved when bridge‐network formation and rheological freedom for alignment are optimally balanced. (e) Comparison of thermal conductivity as a function of total filler loading for this work and previously reported polymer composites based on BN/Al_2_O_3_, Al_2_O_3_‐only, and other hybrid filler systems.

Figure 4a first evaluates the composition dependence of through‐plane thermal conductivity at a fixed total filler loading of 40 vol% by varying the Al_2_O_3_:hBN@Fe_3_O_4_ ratio from 10:0 to 6:4. Before magnetic alignment, the through‐plane thermal conductivity increases monotonically from 1.169 to 1.383 W m^−^^1^ K^−^^1^ as the hBN@Fe_3_O_4_ fraction increases, consistent with the introduction of additional anisotropic conductive contacts into the sphere‐dominant network. After magnetic‐field‐induced alignment, however, the response becomes strongly non‐monotonic: the conductivity changes to 1.169, 1.480, 2.020, 1.618, and 1.626 W m^−^^1^ K^−^^1^ for 10:0, 9:1, 8:2, 7:3, and 6:4, respectively, with the 8:2 composition exhibiting the highest value and the largest alignment‐induced increase. This result demonstrates that the optimal thermal pathway is not obtained by simply maximizing the bridge‐filler content. Instead, the highest performance emerges when the bridge‐filler population is large enough to connect neighboring Al_2_O_3_ particles, yet not so large that viscosity and inter‐filler crowding suppress field‐driven reorientation.

This composition dependence is fully consistent with the structural picture established in Section 2.2. In the 9:1 composite, the hBN@Fe_3_O_4_ fraction is sufficiently low that, even after magnetic alignment, the flakes cannot form a sufficiently dense bridge network between neighboring spherical fillers. By contrast, in the 8:2 composite, a relatively small fraction of aligned hBN@Fe_3_O_4_ flakes is enough to selectively connect adjacent Al_2_O_3_ particles, thereby mitigating point‐contact‐dominated thermal bottlenecks while simultaneously introducing a more continuous and vertically biased heat‐transfer pathway. At still higher hBN@Fe_3_O_4_ fractions, such as 7:3 and 6:4, the number of potential bridges increases, but the actual effectiveness of those bridges declines because the increased viscosity and incomplete reorientation hinder the formation of a coherent through‐plane architecture. A more detailed discussion of this non‐monotonic topology–mobility trade‐off is provided in Note S4.

On the basis of this screening, the 8:2 hybrid ratio was selected as the optimal composition. An additional comparison of hBN@Fe_3_O_4_ fillers with lateral sizes of 5, 18, and 30 µm showed that the 5 µm filler provided the highest through‐plane thermal conductivity and alignment‐induced enhancement under otherwise identical conditions (see Note S5 together with Figure S10 and S11). Accordingly, the optimized 8:2 hybrid composite containing 5 µm hBN@Fe_3_O_4_ was further examined as a function of total filler loading (Figure 4b). For the Al_2_O_3_‐only composites, the through‐plane thermal conductivity increases from 0.226 to 1.957 W m^−^^1^ K^−^^1^ as the filler loading rises from 10 to 45 vol%, reflecting the expected densification of the sphere‐based network. However, the aligned 8:2 hybrid composites exhibit substantially larger gains over the same loading range. At 10, 20, 30, and 40 vol%, the experimentally measured through‐plane thermal conductivities of the sphere‐only composites are 0.2255, 0.3993, 0.6480, and 1.1690 W m^−^^1^ K^−^^1^, whereas those of the hybrid aligned composites increase to 0.2952, 0.6376, 1.1044, and 2.0200 W m^−^^1^ K^−^^1^, corresponding to enhancements of 31, 60, 70, and 73%, respectively. These results show that the thermal benefit of aligned bridge fillers becomes increasingly pronounced as the underlying particulate network becomes denser, because the opportunity for bridge‐mediated relief of contact‐limited bottlenecks grows with filler proximity.

Notably, this enhancement is not maintained at higher filler loading. Although the aligned hybrid composite reaches its highest absolute through‐plane thermal conductivity at 45 vol%, the improvement over the sphere‐only counterpart drops sharply to 12.62%, indicating that the contribution of the aligned bridge‐filler architecture becomes substantially reduced in this highly loaded regime. This tendency becomes clearer when the loading‐dependent changes from 40 to 45 vol% are compared. Over this interval, the thermal conductivity of the sphere‐only composite increases markedly from 1.169 to 1.957 W m^−^^1^ K^−^^1^, and that of the non‐aligned hybrid composite similarly rises from 1.211 to 1.981 W m^−^^1^ K^−^^1^, whereas the aligned hybrid composite shows only a relatively modest increase from 2.020 to 2.204 W m^−^^1^ K^−^^1^. These trends suggest that, at 45 vol%, the conductivity gain is governed primarily by filler‐loading‐induced densification and percolation among closely packed particles, rather than by any further amplification of the aligned bridge effect. This interpretation is fully consistent with the rheological limitation identified in Section 2.2. In the highly loaded regime, the uncured composite becomes sufficiently viscous that the hBN@Fe_3_O_4_ flakes cannot undergo effective field‐driven rotation and stabilization before curing, meaning that the intended bridge‐alignment architecture can no longer be fully expressed. Thus, the loading dependence in Figure 4b highlights an important design rule: the thermal advantage of magnetically aligned bridge fillers is maximized not at the highest possible filler loading, but within a processable window where directional bridge formation remains possible.

Figure 4c,d further quantifies the directional character of this optimized hybrid architecture by comparing through‐plane and in‐plane thermal conductivity and by plotting the corresponding anisotropy ratio of the aligned 8:2 composites as a function of filler loading. These data indicate that the aligned bridge‐filler strategy does not simply increase overall conductivity isotropically; rather, it preferentially reinforces heat transport along the through‐plane direction, which is the most relevant pathway for underfill‐type thermal management. In the context of the present materials design, this distinction is crucial, because the objective is not maximal lateral heat spreading but selective mitigation of vertical heat‐extraction bottlenecks in electrically insulating packaging composites.

The comparative plot in Figure 4e further places the thermal performance of the optimized composite in the context of previously reported polymer composites [64, 65, 66, 67, 68, 69, 70, 71, 72]. Compared with Al_2_O_3_‐only systems, which generally exhibit limited thermal conductivity because of point‐contact‐dominated heat‐transfer pathways, the present aligned Al_2_O_3_/hBN@Fe_3_O_4_ composite achieves a substantially higher thermal conductivity at a moderate total filler loading. Its performance also lies within the upper range of reported BN/Al_2_O_3_‐based composites and exceeds that of many other hybrid filler systems at comparable or higher filler contents. This comparison indicates that the enhanced thermal transport does not arise solely from increasing the total filler fraction, but from the efficient utilization of a relatively small population of magnetically aligned hBN bridges to reorganize the sphere‐dominant thermal network. Furthermore, a detailed comparison of the present work with representative magnetically aligned BN‐based polymer composites is summarized in Table S1.

Taken together, Figure 4 shows that through‐plane thermal transport in the present system is governed by a topology‐optimized balance among bridge‐filler fraction, magnetic alignability, and composite processability. The 8:2 composition at 40 vol% total loading represents the regime in which these competing requirements are most effectively reconciled, yielding a through‐plane thermal conductivity of 2.02 W m^−^^1^ K^−^^1^ and a 72.8% improvement over the corresponding Al_2_O_3_‐only composite. More broadly, these results support the central thesis of this work: contact‐limited thermal bottlenecks in sphere‐dominant polymer composites can be mitigated not merely by adding more conductive filler, but by engineering a process‐compatible, directionally programmable bridge network that selectively reorganizes heat‐flow continuity. The mechanistic origin of this effect is examined further in the following section using finite‐element modeling.

### 2.4 FEM‐Guided Mechanistic Analysis of Thermal‐Bottleneck Mitigation

The experimental results in Sections 2.2 and 2.3 indicate that the aligned Al_2_O_3_/hBN@Fe_3_O_4_ hybrid architecture outperforms the sphere‐only composite not merely because it contains an additional anisotropic filler, but because it reorganizes heat‐flow continuity across the composite thickness. To clarify the physical origin of this improvement, finite‐element method (FEM) was carried out to compare three representative microstructures: sphere‐only, sphere + flake non‐aligned, and sphere + flake aligned composites (Figure 5). In this way, the simulation was designed not simply to reproduce a thermal‐conductivity value, but to reveal how bridge formation and directional alignment alter the topology of heat transport in particle‐filled polymer composites.

**FIGURE 5:**
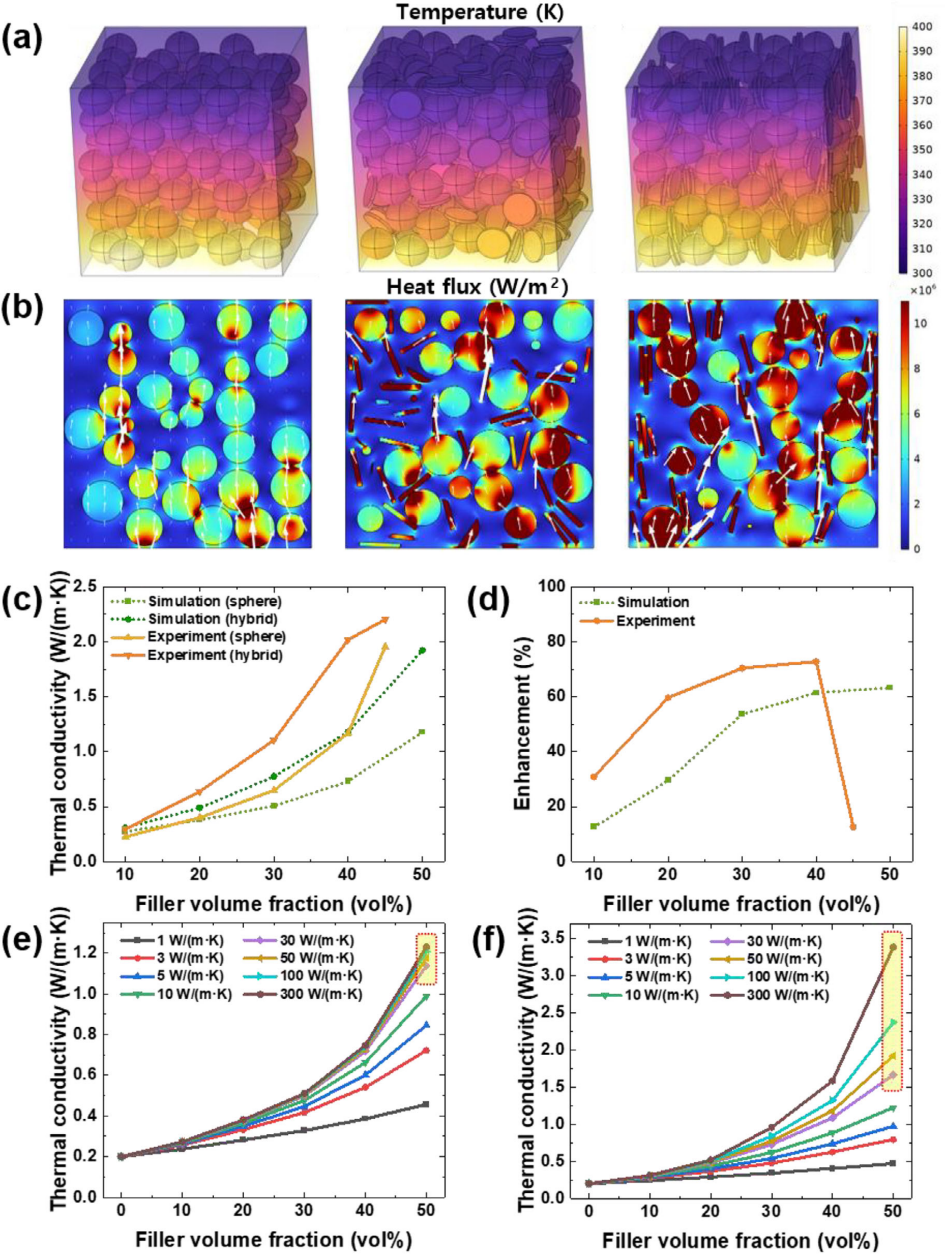
FEM‐guided mechanistic analysis of thermal‐bottleneck mitigation in hybrid composites. (a) FEM‐calculated temperature distributions for sphere‐only, sphere + flake non‐aligned, and sphere + flake aligned composite structures at a total filler loading of 40 vol%. (b) FEM‐calculated heat‐flux distributions on the y–z cross‐sectional plane for the corresponding structures, showing heat‐flux concentration in the sphere‐only network and its redistribution in the hybrid aligned network. (c) Comparison of experimentally measured and FEM‐calculated through‐plane thermal conductivity for sphere‐only and hybrid aligned composites (Al_2_O_3_:hBN@Fe_3_O_4_ = 8:2). (d) Comparison of experimentally measured and FEM‐calculated conductivity enhancement from the sphere‐only to the hybrid aligned structure. (e) FEM‐calculated effective thermal conductivity of sphere‐only composites as a function of intrinsic filler thermal conductivity, showing saturation‐like behavior associated with contact‐limited heat transport. (f) FEM‐calculated effective thermal conductivity of sphere + aligned flake hybrid composites as a function of intrinsic filler thermal conductivity, showing mitigation of the thermal bottleneck through bridge‐mediated and directionally organized heat pathways.

The FEM model was constructed using a 30 µm cubic domain containing randomly packed fillers, where spherical fillers were represented with a diameter of 5 µm and flake fillers with a lateral size of 5 µm and a thickness of 0.5 µm. For the structure‐comparison analysis in Figure 5a,b, the total filler loading was fixed at 40 vol%, matching the experimentally optimized condition, and the lower and upper surfaces were set to 400 and 300 K, respectively, with the remaining boundaries treated as adiabatic. An interfacial thermal resistance of 2.07 × 10^−8^ m^2^K W^−1^ was introduced at the filler–matrix interfaces, while the matrix and filler thermal conductivities were set to 0.2 and 50 W m^−^^1^ K^−^^1^, respectively [73]. Additional simulation geometries covering 10–50 vol% filler loading are provided in Figure S12, and model validation against commercial SiO_2_/epoxy and Al_2_O_3_/epoxy composites is shown in Figure S13. A more detailed description of the modeling rationale and its relation to effective‐medium behavior is provided in Note S6.

The simulated temperature and heat‐flux distributions clearly distinguish the three structural cases (Figure 5a,b). In the sphere‐only composite, heat flux becomes strongly concentrated at localized contacts between neighboring spherical fillers. This behavior reflects the intrinsic limitation of sphere‐dominant particulate networks, in which the real contact area between adjacent fillers is highly restricted and thermal transport must pass through geometrically constricted junctions. As a result, the local heat path is repeatedly interrupted by point‐contact‐mediated resistance, giving rise to a pronounced thermal bottleneck. The hybrid non‐aligned structure partially relieves this limitation by introducing flake fillers that can bridge some neighboring particles, but the resulting pathways remain spatially discontinuous because the orientation of the flakes is still stochastic. By contrast, in the hybrid aligned structure, the flake bridges are preferentially organized along the through‐plane direction, creating more continuous heat‐flow channels and reducing the concentration of heat flux at isolated junctions. In this sense, magnetic alignment does not merely change filler orientation; it changes the topology by which thermal resistance is distributed throughout the composite.

This mechanistic interpretation is consistent with the quantitative comparison between experiment and simulation (Figure 5c,d). The FEM results reproduce the same overall trend observed experimentally, namely that the hybrid aligned composites generate progressively larger through‐plane conductivity gains than the sphere‐only composites over the 10–40 vol% range. In the simulations, the conductivity enhancement of the hybrid aligned structure relative to the sphere‐only structure reaches 13, 30, 54, and 61% at 10, 20, 30, and 40 vol%, respectively. Experimentally, the corresponding enhancements are even larger, namely 31, 60, 70, and 73%. Although the absolute values are not identical, the close agreement in trend confirms that the dominant origin of the thermal improvement lies in bridge‐enabled and directionally organized network formation rather than in a fitting artifact. The stronger enhancement observed experimentally may reflect microstructural details not fully captured in the idealized geometric model, but the simulation robustly reproduces the essential transition from contact‐limited heat transport to bridge‐assisted heat‐flow continuity.

An additional insight emerges when the intrinsic thermal conductivity of the filler phase is systematically varied in the simulations (Figure 5e,f). For the sphere‐only architecture, the effective composite thermal conductivity initially increases with increasing filler conductivity, but the improvement gradually saturates once the filler conductivity exceeds approximately 30 W m^−^^1^ K^−^^1^. This saturation indicates that, beyond a certain point, the thermal conductivity of the filler itself is no longer the dominant limitation. Instead, the composite becomes governed by inter‐filler contact constriction and interfacial resistance, meaning that further improvement in filler intrinsic conductivity cannot be efficiently translated into macroscopic composite performance. A similar saturation tendency is also predicted by the Lewis–Nielsen model (Figure S14), further supporting the interpretation that sphere‐only systems are fundamentally bottleneck‐limited [74, 75].

By contrast, the sphere + aligned flake hybrid architecture shows a markedly different response. In this case, the effective thermal conductivity continues to increase with increasing filler conductivity, and at a filler conductivity of 300 W m^−^^1^ K^−^^1^ and a total filler loading of 50 vol%, the hybrid model reaches an effective thermal conductivity of approximately 3.39 W m^−^^1^ K^−^^1^. This behavior indicates that once the bridge network is made sufficiently continuous and directionally coherent, the composite can make more efficient use of the intrinsic thermal transport capability of the filler phase. Put differently, the aligned bridge architecture shifts the system away from a contact‐bottleneck regime and toward a transport regime in which improvements in filler properties can be more directly converted into composite‐level performance.

Overall, Figure 5 provides a physics‐based explanation for the experimentally observed optimum identified in Section 2.3. The hybrid aligned structure is superior not simply because it contains hBN@Fe_3_O_4_ flakes, but because those flakes selectively bridge neighboring spherical fillers and redistribute heat flux away from discrete point‐contact bottlenecks. In this way, magnetic alignment transforms the hybrid composite from a particulate assembly governed by contact limitation into a more continuous through‐plane heat‐transport network. More broadly, these simulations support the central thesis of this work: in sphere‐dominant, electrically insulating polymer composites, improving thermal management requires not only conductive fillers, but deliberate control over how those fillers connect, bridge, and orient across multiple length scales.

### 2.5 Electrical Insulation and Macroscopic Heat‐Dissipation Performance of the Optimized Hybrid Architecture

For underfill‐relevant thermal‐management materials, however, high thermal conductivity alone is not sufficient; the composite must also retain electrically insulating behavior and translate its improved internal heat pathway into macroscopic heat‐dissipation performance. Figure 6 therefore evaluates the optimized 8:2 hybrid system from these two complementary perspectives by comparing its electrical‐insulation characteristics with those of neat epoxy and an Al_2_O_3_‐only composite, and by examining its thermal response in a package‐level configuration. The dielectric constant results in Figure 6a indicate that the three materials maintain relatively stable dielectric responses over the investigated frequency range. At 1 Hz, the dielectric constants of neat epoxy, the 40 vol% Al_2_O_3_‐only composite, and the aligned 40 vol% hybrid composite are 3.18, 5.89, and 4.89, respectively, while the corresponding values near 1 MHz are approximately 3.00, 5.67, and 4.53. All samples show a slight decrease in dielectric constant with increasing frequency. This behavior is consistent with the frequency dependence of interfacial polarization: at lower frequencies, dipolar and interfacial charges can respond more effectively to the applied field, whereas at higher frequencies the polarization response becomes progressively restricted [76]. Notably, the aligned hybrid composite exhibits a lower dielectric constant than the Al_2_O_3_‐only composite throughout the measured frequency range. Importantly, despite this expected trend, the dielectric constant remains comparatively moderate and only weakly frequency‐dependent, indicating that the introduction of aligned bridge fillers does not compromise the electrically insulating character required for packaging‐related applications. A more detailed discussion of the dielectric interpretation is provided in Note S7.

**FIGURE 6:**
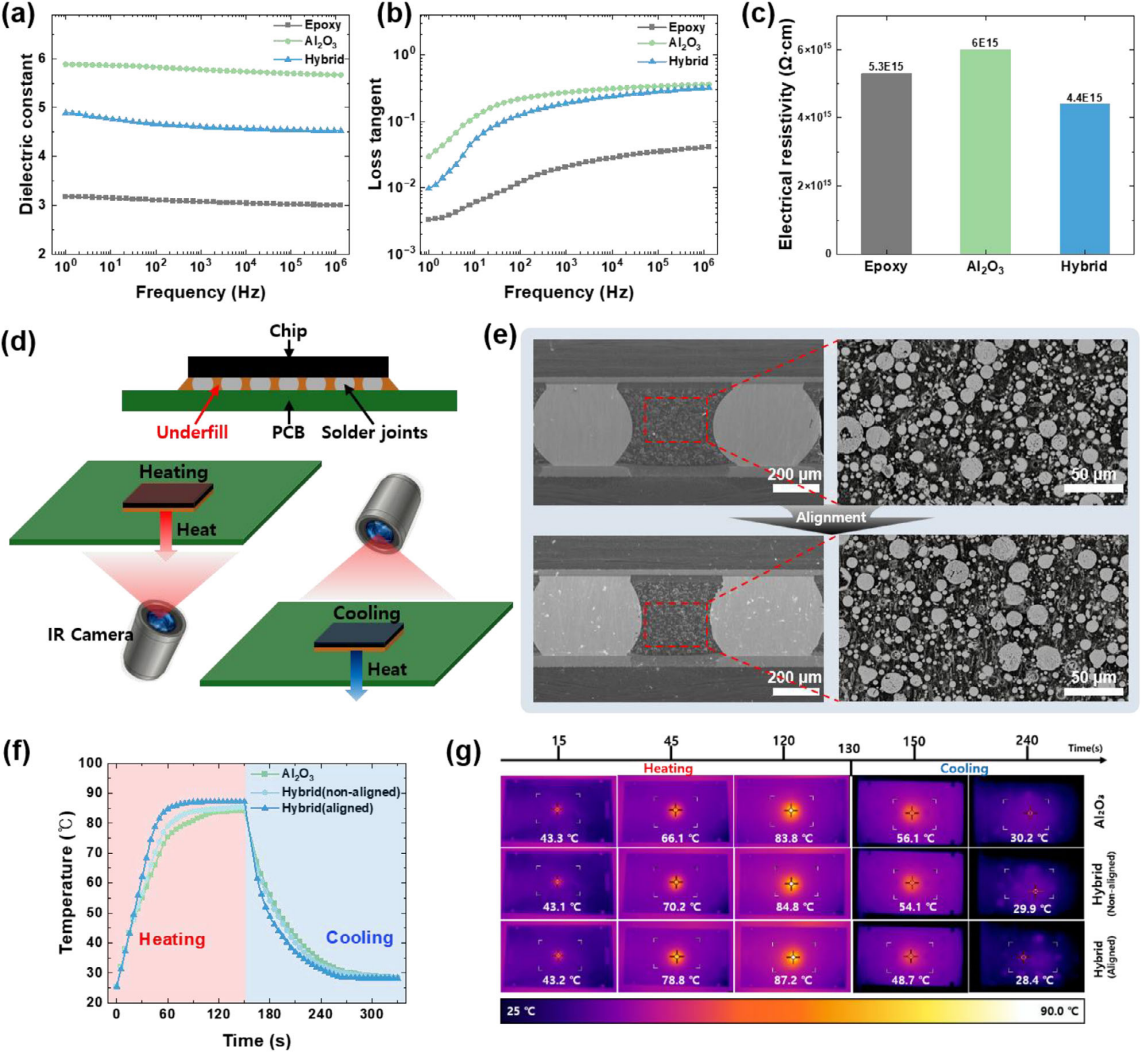
Electrical insulation and package‐level thermal‐management performance of the optimized hybrid composite. (a) Frequency‐dependent dielectric constant and (b) loss tangent of neat epoxy, the Al_2_O_3_/epoxy composite containing 40 vol% Al_2_O_3_, and the magnetically aligned Al_2_O_3_/hBN@Fe_3_O_4_/epoxy composite containing 40 vol% total filler at an Al_2_O_3_:hBN@Fe_3_O_4_ volume ratio of 8:2. (c) Volume resistivity of the corresponding neat epoxy, Al_2_O_3_/epoxy, and aligned hybrid composites. (d) Schematic illustration of the bump‐bonded semiconductor package containing the composite underfill and the infrared‐camera configurations used to monitor its thermal response during heating and cooling. (e) Cross‐sectional SEM images of the fabricated semiconductor packages containing non‐aligned and magnetically aligned hybrid underfills. (f) Time‐dependent temperature profiles of the packaged samples during the heating and cooling cycle. (g) Corresponding time‐resolved infrared thermal images acquired during heating and subsequent cooling.

A similar tendency is observed for dielectric loss (Figure 6b). At 1 Hz, the loss tangent values of neat epoxy, the Al_2_O_3_‐only composite, and the aligned hybrid composite are approximately 0.003, 0.029, and 0.010, respectively. The loss tangent gradually increases with frequency, reaching approximately 0.040, 0.355, and 0.314 near 1 MHz. Although the aligned hybrid composite exhibits greater dielectric loss than neat epoxy, its values remain consistently lower than those of the Al_2_O_3_‐only composite over the investigated frequency range. These results suggest that although the addition of hybrid fillers introduces some increase in dielectric dissipation, the resulting composites still remain within a practically acceptable range for electrically insulating thermal‐management materials. In other words, the bridge‐enabled thermal‐network design improves heat transport without imposing a severe dielectric penalty.

The volume‐resistivity results in Figure 6c further confirm the bulk electrical insulation of the composite system. Neat epoxy, the Al_2_O_3_‐only composite, and the aligned hybrid composite exhibit volume resistivities of approximately 5.3 × 10^15^, 6.0 × 10^15^, and 4.4 × 10^15^ Ω · cm, respectively. Although the hybrid composite shows a modestly lower value, its volume resistivity remains on the order of 10^15^ Ω · cm, demonstrating that the electrically insulating character is retained despite the incorporation of Fe_3_O_4_‐functionalized hBN bridge fillers.

To assess whether the improved internal heat‐transport network also manifests at the package level, the Al_2_O_3_‐only, hybrid non‐aligned, and hybrid aligned composites were applied as underfills in bump‐bonded chip packages, and their transient thermal‐transfer behavior was evaluated during heating and cooling (Figure 6d–g). Cross‐sectional SEM images in Figure 6e confirm that the underfills were successfully introduced between the chip and PCB without visible void formation. The hBN@Fe_3_O_4_ fillers in the aligned underfill also retain a preferential through‐plane orientation within the confined spaces between adjacent bumps. Detailed package configurations, photographs of the fabricated samples (Figure S15), and thermal‐measurement procedures are provided in Note S7.

During heating, an external 100 °C heater was applied to the chip for 150 s, while the temperature of the opposite PCB surface was monitored by IR thermal imaging. All samples exhibit progressive PCB‐surface heating with time, but the heating rate depends strongly on the underfill architecture. At 45 s, the PCB temperatures of the Al_2_O_3_‐only, hybrid non‐aligned, and hybrid aligned packages are 66.1, 70.2, and 78.8 °C, respectively. After removal of the heater, the chip temperature was monitored during natural cooling, with the hybrid aligned package exhibiting the fastest temperature decrease. At 30 s of cooling, the chip temperatures are 56.1, 54.1, and 48.7 °C for the Al_2_O_3_‐only, hybrid non‐aligned, and hybrid aligned packages, respectively.

This ranking directly mirrors the through‐plane thermal‐conductivity trend established in Section 2.3, indicating that the bridge‐enabled and magnetically aligned filler architecture improves not only intrinsic material metrics but also the practical rate at which heat can be transferred between the chip and PCB through the underfill layer. In the present package geometry, the faster PCB‐temperature rise during heating and the more rapid chip‐temperature decrease during cooling both indicate enhanced through‐plane heat transfer. The hybrid aligned composite therefore behaves as the most effective vertical heat‐transfer medium among the tested underfills. From a design standpoint, this result is important because it shows that the thermal‐network reorganization identified microscopically and validated numerically is preserved after integration into a bump‐bonded semiconductor package.

More broadly, Figure 6 demonstrates that the optimized Al_2_O_3_/hBN@Fe_3_O_4_ hybrid architecture reconciles two requirements that are often difficult to achieve simultaneously in electrically insulating packaging composites: enhanced through‐plane heat dissipation and stable dielectric behavior. The present system should therefore be viewed not merely as a high‐thermal‐conductivity filler formulation, but as a directionally engineered insulating composite in which bridge‐network design, magnetic programmability, and rheology‐mediated processability collectively translate into application‐relevant thermal performance.

Additionally, the magnetic flux density applied in this study (0.678 T) is substantially lower than the magnetic‐field range reported to affect standard CMOS structures only weakly. Previous studies showed that subthreshold CMOS transistors remained largely insensitive even above 11 T, and that CMOS thin films and contacts exhibited mostly negligible magnetoresistance up to 6 T [77, 78]. Therefore, we expect that the magnetic flux density used in this work is considered sufficiently low for practical silicon‐based integrated electronics and conventional semiconductor packaging assemblies. While a full underfill qualification would require additional package‐specific metrics, the results here clearly establish the functional relevance of the aligned bridge‐filler concept for advanced packaging‐oriented thermal‐management materials.

## 3 Conclusion

In this study, we introduced a magnetically programmable bridge‐filler architecture to mitigate contact‐limited thermal bottlenecks in electrically insulating polymer composites. The central concept was to move beyond conventional sphere‐only thermal filler systems, in which heat transport is constrained by discontinuous point contacts between neighboring particles, and instead create a directionally organized hybrid network in which Fe_3_O_4_‐functionalized hexagonal boron nitride (hBN@Fe_3_O_4_) flakes act as magnetically addressable bridges between spherical Al_2_O_3_ fillers. Through this design, the processability advantage of a sphere‐dominant composite could be combined with the directional heat‐transport capability of anisotropic bridge fillers. The results consistently showed that the thermal performance of the composite was governed not simply by filler loading or filler intrinsic conductivity, but by how effectively the hybrid filler network could be reorganized under processing‐accessible conditions. At a fixed total filler loading of 40 vol%, the Al_2_O_3_:hBN@Fe_3_O_4_ ratio of 8:2 provided the most effective balance between bridge‐network formation and rheological freedom for magnetic alignment, yielding a through‐plane thermal conductivity of 2.02 W m^−^^1^ K^−^^1^, which corresponds to a 72.8% enhancement over the corresponding Al_2_O_3_‐only composite. By contrast, further increasing the hBN@Fe_3_O_4_ fraction did not produce additional gains because the higher viscosity increasingly suppressed field‐induced reorientation of the bridge fillers. These results establish that the optimal design of hybrid thermal composites must be understood as a topology–mobility balance rather than as a simple maximization of anisotropic filler content.

Finite‐element modeling further clarified the physical origin of this improvement. In sphere‐only composites, heat flux was strongly concentrated at localized interparticle contacts, leading to pronounced thermal bottlenecks and saturation‐like behavior even when the intrinsic conductivity of the filler phase was increased. In contrast, the hybrid aligned architecture redistributed heat flow through more continuous bridge‐mediated pathways and reduced the severity of point‐contact‐dominated thermal constrictions. The agreement between experiment, simulation, and effective‐medium analysis therefore indicates that the observed performance gain originates from a structural reorganization of heat‐flow continuity rather than from a merely additive filler effect. Importantly, the optimized hybrid composite preserved bulk electrical insulation while exhibiting improved package‐level thermal‐transfer performance. The aligned hybrid composite maintained a volume resistivity of 4.4 × 10^1^ ^5^ Ω cm and exhibited lower dielectric constant and dielectric loss than the Al_2_O_3_‐only composite over the investigated frequency range. In bump‐bonded semiconductor packages, the aligned hybrid underfill exhibited improved thermal‐transfer performance under practical package‐level conditions. These results indicate that the bridge‐enabled and field‐directed thermal network can enhance through‐plane heat transport without sacrificing the insulating characteristics required for packaging‐oriented thermal‐management materials. Overall, this work establishes a generalizable materials design principle for thermally conductive polymer composites: thermal bottlenecks in sphere‐dominant insulating systems can be alleviated not only by increasing filler content, but by engineering how fillers bridge, connect, and orient across multiple length scales. Although the present study focused on a specific Al_2_O_3_/hBN@Fe_3_O_4_/epoxy combination, the underlying concept of bridge‐enabled and field‐directed thermal pathway engineering should be extendable to other filler chemistries, geometries, and magnetic‐response schemes. In this sense, the present system should be viewed not merely as an optimized filler formulation, but as a proof of a broader design strategy for directionally efficient, electrically insulating thermal‐management composites and promising packaging‐oriented materials.

## 4 Methods

### 4.1 Materials

Hexagonal boron nitride (hBN) flakes with nominal lateral sizes of approximately 5, 18, and 30 µm (purity 99.8%, Denka) and spherical alumina (Al_2_O_3_) powder (purity 99.8%, average particle size ≈ 5 µm, Denka) were used as thermally conductive fillers. Deionized (DI) water was obtained from a Milli‐Q Direct 8 water purification system (Merck Millipore, USA). Ethanol (99.9%, Sigma‐Aldrich) was used as a co‐solvent and washing solvent. Dopamine hydrochloride (≥98%, Sigma‐Aldrich), tris(hydroxymethyl)aminomethane hydrochloride (Tris–HCl, ≥99%, Sigma‐Aldrich), sodium hydroxide solution (NaOH, 1 M, Samchun Chemicals), ammonia solution (NH_4_OH, 25 wt%, Sigma‐Aldrich), iron(II) chloride tetrahydrate (FeCl_2_·4H_2_O, ≥99%, Daejung Chemicals), and iron(III) chloride hexahydrate (FeCl_3_·6H_2_O, ≥98%, Daejung Chemicals) were used as received. Permanent neodymium magnets (50 × 50 × 10 mm, NS Magnet) were used for magnetic alignment. Ultra‐high‐purity nitrogen gas (99.999%, JC Gas, Korea) was employed during Fe_3_O_4_ co‐precipitation. For composite fabrication, bisphenol‐F epoxy resin (DGEBF, 99.5%, Kukdo Chemical), isophorone diamine (IPDA, 99.8%, Sigma‐Aldrich), and butyl glycidyl ether (BGE, ≥98%, Tokyo Chemical Industry Co., Ltd.) were used as the matrix resin, curing agent, and reactive diluent, respectively. The semiconductor chips and printed circuit boards (PCBs) used for package fabrication were purchased from Amkor Technology and Korea Circuit Co., Ltd., respectively.

### 4.2 Preparation of PDA‐Coated hBN

To introduce an interfacial layer for subsequent magnetic functionalization, hBN flakes were first coated with polydopamine (PDA). A Tris buffer solution was prepared by dissolving 0.355 g of Tris–HCl (≈2.25 mmol) in 225 mL of DI water, and the pH was adjusted to 8.5 by dropwise addition of NaOH solution under stirring. Subsequently, 5 g of hBN flakes were dispersed in the buffer together with 75 mL of ethanol and ultrasonicated for 30 min. Dopamine hydrochloride (0.5 g, corresponding to 10 wt% of the hBN mass) was then added, and the suspension was stirred at room temperature for 24 h to allow oxidative self‐polymerization of dopamine on the hBN surface. The resulting PDA‐coated hBN was collected by centrifugation, washed several times with ethanol and DI water, and dried at 80 °C for 10 h.

### 4.3 Synthesis of Fe_3_O_4_‐Functionalized hBN (hBN@Fe_3_O_4_)

Fe_3_O_4_‐functionalized hBN was prepared by co‐precipitation of iron ions on PDA‐coated hBN. Briefly, 5 g of PDA‐coated hBN was dispersed in 300 mL of DI water and ultrasonicated for 30 min. Iron salts, namely 1.0 g of FeCl_2_·4H_2_O and 2.5 g of FeCl_3_·6H_2_O, were then added to the dispersion, corresponding to an Fe^2^^+^:Fe^3^^+^ molar ratio of 1:2. The mixture was stirred at 50°C for 5 h under a nitrogen atmosphere to promote adsorption of iron ions onto the PDA layer. The temperature was then increased to 65°C, and NH_4_OH solution was added dropwise until the pH reached approximately 11, thereby initiating Fe_3_O_4_ co‐precipitation. The suspension was further stirred at 600 rpm for 2 h. The resulting Fe_3_O_4_‐decorated PDA‐coated hBN was collected by repeated washing with ethanol and DI water and dried at 80°C for 12 h. For simplicity, this filler is denoted as hBN@Fe_3_O_4_ throughout the manuscript.

### 4.4 Fabrication of Al_2_O_3_/hBN@Fe_3_O_4_/Epoxy Composites and Magnetic Alignment

Hybrid composites were fabricated using spherical Al_2_O_3_ and hBN@Fe_3_O_4_ as fillers in a DGEBF‐based epoxy matrix. BGE was first blended with DGEBF at 10 wt% relative to the epoxy resin to reduce Scosity. The desired amounts of Al_2_O_3_ and hBN@Fe_3_O_4_ were then added and mixed using a planetary mixer until homogeneous dispersion was obtained. IPDA was subsequently introduced at 25 wt% relative to the total resin content (DGEBF + BGE), followed by additional mixing. To screen the effect of hybrid filler composition, the total filler loading was fixed at 40 vol%, and the Al_2_O_3_:hBN@Fe_3_O_4_ volume ratio was varied as 10:0, 9:1, 8:2, 7:3, and 6:4. On the basis of this screening, the 8:2 composition was selected as the optimal hybrid ratio. To examine filler‐loading dependence, Al_2_O_3_‐only composites and Al_2_O_3_:hBN@Fe_3_O_4_ (8:2) composites were then prepared at total filler loadings of 10, 20, 30, 40, and 45 vol%. For rheological and alignment analyses, representative compositions of 10:0, 8:2, and 6:4 at 40 vol% total filler loading were comparatively investigated. The uncured mixtures were cast into Teflon molds. For magnetic alignment, the mold was placed in a custom‐built setup equipped with NdFeB magnets arranged to generate a vertical magnetic field of approximately 0.38 T. The magnetic flux density experienced by the sample at a 4 mm separation from each magnet was estimated to be approximately 0.678 T. The samples were kept under the magnetic field for ≈12 h during the pre‐curing stage to allow directional reorientation of hBN@Fe_3_O_4_ within the viscous matrix. The samples were then thermally cured at 80 °C for 2 h and subsequently at 120 °C for 2 h. For comparison, non‐aligned samples were prepared under otherwise identical conditions but without magnetic‐field exposure.

### 4.5 Structural, Magnetic, and Rheological Characterization

The morphology and microstructure of the fillers and composites were analyzed using field‐emission scanning electron microscopy coupled with energy‐dispersive X‐ray spectroscopy (FE‐SEM/EDS; JSM‐IT800, JEOL Ltd., Japan). Crystal structures were characterized by X‐ray diffraction (XRD; SmartLab, Rigaku Corporation, Japan). Chemical bonding states were investigated by Fourier‐transform infrared spectroscopy (FT‐IR; Nicolet iS50, Thermo Fisher Scientific, USA) and X‐ray photoelectron spectroscopy (XPS; K‐Alpha, Thermo Fisher Scientific, USA). The Fe contents of hBN@Fe_3_O_4_ prepared with different iron‐precursor feed amounts were quantified using inductively coupled plasma optical emission spectrometry (ICP‐OES; Agilent 5110, Agilent Technologies, USA). The corresponding Fe_3_O_4_ contents were calculated from the measured Fe concentrations using the stoichiometric Fe fraction of Fe_3_O_4_. Magnetic properties were measured using a vibrating sample magnetometer (VSM; 8607, Lake Shore Cryotronics, USA) at room temperature. Rheological behavior of the uncured composites was evaluated using a rheometer (MCR 302e, Anton Paar GmbH, Austria) over a shear‐rate range of 1–200 s^−^^1^. Cross‐sectional filler orientation before and after magnetic alignment was evaluated from SEM images and their corresponding fast Fourier transform (FFT) patterns. Additional image‐analysis details are provided in Methods S1.

### 4.6 Thermal, Dielectric, and Infrared Heating Measurements

Through‐plane thermal diffusivity (α) was measured using a laser flash analyzer (LFA 467 HyperFlash, NETZSCH‐Gerätebau GmbH, Germany). The density (ρ) of the composites was determined by the Archimedes method using an analytical balance equipped with a density kit (XPR205DR with Density Kit, METTLER TOLEDO, Switzerland). The specific heat capacity (*C_p_*) was measured using differential scanning calorimetry (DSC 214, NETZSCH, Germany). The through‐plane thermal conductivity (κ) was then calculated according to κ  =  αρ*C_p_*. In‐plane and through‐plane thermal conductivity were compared for the aligned Al_2_O_3_/hBN@Fe_3_O_4_ (8:2) composites as a function of total filler loading. Dielectric properties were measured using a broadband dielectric spectrometer (Concept 80, Novocontrol Technologies, Germany). The dielectric constant and loss tangent were recorded over the frequency range from 1 Hz to 1 MHz for neat epoxy, the Al_2_O_3_/epoxy composite containing 40 vol% Al_2_O_3_, and the magnetically aligned Al_2_O_3_/hBN@Fe_3_O_4_/epoxy composite containing 40 vol% total filler at an Al_2_O_3_:hBN@Fe_3_O_4_ volume ratio of 8:2. Volume resistivity was measured for the corresponding samples in accordance with ASTM D257.

Package‐level thermal‐transfer behavior was evaluated using bump‐bonded chip packages containing Al_2_O_3_‐only, hybrid non‐aligned, or hybrid aligned underfills. During heating, a 100°C external heater was applied to the chip for 150 s, while the temperature of the opposite PCB surface was monitored by infrared thermal imaging. After removal of the heater, the chip temperature was monitored during 180 s of natural cooling.

## Author Contributions

**Chan‐Jae Lee**: methodology, funding acquisition. **Sabina Ghorsaine**: conceptualization, validation, data curation. **Mooho Lee**: funding acquisition, validation. **In Kim**: funding acquisition, validation. **Yeonwook Jeong**: methodology, software, formal analysis, writing – original draft. **Jong‐Woong Kim**: conceptualization, validation, supervision, project administration, funding acquisition, writing – review and editing. **Hyejeong Lee**: methodology, investigation, funding acquisition. **Ginam Kim**: writing – review and editing, conceptualization.

## Conflicts of Interest

The authors declare no conflict of interest.

## Supporting information




**Supporting File**: smll75261‐sup‐0001‐SuppMat.docx.

## Data Availability

The data that support the findings of this study are available from the corresponding author upon reasonable request.
