# Supplementary material for: Mitigating Contact‐Constricted Thermal Bottlenecks by Magnetically Programmable hBN Bridge Fillers in Al2O3/Epoxy Composites
Source: Small. 2026 Aug 18;22(59):e75261. doi: 10.1002/smll.75261 (PMC13690618; doi:10.1002/smll.75261)
Supplement: Supplementary file 1 [file SMLL-22-e75261-s001.docx]

Supporting Information

Mitigating Contact-Constricted Thermal Bottlenecks by Magnetically Programmable hBN Bridge Fillers in Al₂O₃/Epoxy Composites

Yeonwook Jeong^#^, Sabina Ghorsaine^#^, Chan-Jae Lee, In Kim, Mooho Lee, Hyejeong Lee, Ginam Kim^*^, and Jong-Woong Kim^*^

**Supporting Note S1. Interfacial Design Rationale for Polydopamine-Assisted Fe₃O₄ Growth on hBN and Preservation of Bridge-Filler Geometry**

In the present hybrid-filler design, the role of hexagonal boron nitride (hBN) is not merely to serve as an additional thermally conductive component, but to function as a geometrically effective bridge filler capable of connecting neighboring spherical Al₂O₃ particles ^[1, 2]^. This function requires that the platelet morphology of hBN be preserved during magnetic functionalization. If surface modification were to induce severe particle overgrowth, aggregation, or fragmentation, the flake would lose its ability to span adjacent spheres and to generate extended interfacial contacts favorable for phonon transport. Therefore, the interfacial chemistry used for magnetic activation must simultaneously satisfy two conditions: efficient immobilization of magnetic domains and retention of the anisotropic flake geometry.

Polydopamine (PDA) provides an effective interfacial platform for this purpose. Because pristine hBN is chemically inert and has limited affinity for direct inorganic deposition, PDA was introduced as an adhesive coating layer containing catechol- and amine-rich functional groups. These functionalities can coordinate iron ions and facilitate heterogeneous nucleation of Fe₃O₄ during the subsequent co-precipitation process. In this way, PDA acts as a chemically active interlayer that couples the hBN surface to Fe₃O₄ formation without requiring harsh surface oxidation or structural disruption of the parent flake ^[3]^. In addition, the polar functionalities of PDA can improve the interfacial compatibility between the functionalized filler and the epoxy matrix, thereby reducing poorly bonded interfacial regions and the associated thermal resistance ^[4]^.

The supporting microscopy and spectroscopy results are consistent with this interfacial design rationale. As shown in **Figure S1**, the starting fillers exhibit clearly distinct morphologies, namely spherical Al₂O₃ particles and platelet-like hBN flakes. After surface functionalization, the hBN flakes retain their overall platelet geometry, while nanoscale Fe-containing domains are distributed over the flake surface (**Figure S2**) ^[3]^. The high-resolution XPS spectra in **Figure S3** further support the coexistence of hBN-derived bonding states, PDA-derived organic functionalities, and oxygen-containing species associated with the interfacial coating and iron oxide formation. The amount of PDA deposited on the hBN surface was further quantified by thermogravimetric analysis, as shown in **Figure S4a**. During heating from room temperature to 700 °C, PDA progressively decomposed, with its decomposition essentially completed near 600 °C. The residual mass of hBN@PDA decreased to 96.82%, indicating that the prepared hBN@PDA contained approximately 3.18 wt% PDA.

To evaluate whether the PDA coating adversely affected thermal transport, pristine hBN and hBN@PDA were separately incorporated into the epoxy matrix at an identical filler loading of 20 vol%. As shown in **Figure S4b**, the thermal conductivities of the pristine hBN/epoxy and hBN@PDA/epoxy composites were 0.549 and 0.571 W m⁻¹ K⁻¹, respectively. The slightly higher thermal conductivity of the hBN@PDA/epoxy composite indicates that the PDA coating at approximately 3.18 wt% did not act as a dominant thermal barrier. Instead, the result supports the beneficial effect of PDA on filler–epoxy interfacial compatibility and thermal transport across the interface. Although an excessively thick PDA coating could increase the thickness of the thermally resistive organic interlayer and hinder heat transfer, the coating level employed in the present study improved the filler–matrix interface without adversely affecting the composite thermal conductivity. These results collectively indicate that magnetic functionalization occurs through surface-mediated decoration rather than bulk structural transformation of hBN.

From a transport-design perspective, this distinction is important. The Fe₃O₄ phase is not introduced as the principal thermally conductive network former; instead, it provides magnetic responsiveness that enables hBN to be actively reoriented during composite processing. Accordingly, the functional contribution of Fe₃O₄ is best understood as a magnetic programming handle, whereas the geometrical and thermally conductive contribution is primarily retained by the hBN flake itself. Meanwhile, the thin PDA coating serves as an interfacial coupling layer that enables Fe₃O₄ immobilization while maintaining favorable compatibility with the epoxy matrix. This separation of roles is central to the present materials strategy, because it allows a small amount of magnetically addressable anisotropic filler to reorganize heat-flow connectivity within a sphere-dominant and processable composite matrix.

**Supporting Note S2. Optimization of Fe₃O₄ Loading on hBN for Magnetic Alignment and Through-Plane Thermal Transport**

The Fe₃O₄ loading on hBN must be optimized to provide sufficient magnetic responsiveness without compromising the intrinsic heat-transport capability of the hBN platelets. Accordingly, four fillers, denoted as hBN@Fe₃O₄–0.5, hBN@Fe₃O₄–2.5, hBN@Fe₃O₄–5, and hBN@Fe₃O₄–10, were prepared using different iron-precursor feed amounts, as indicated by their numerical suffixes. Among these, hBN@Fe₃O₄–2.5 corresponds to the formulation used in the main study.

As shown in **Figure S5**, hBN@Fe₃O₄–0.5 exhibited a small amount of sparsely distributed, island-like Fe₃O₄ on the hBN surface. hBN@Fe₃O₄–2.5 retained a similar island-like morphology but showed visibly greater Fe₃O₄ coverage. In contrast, hBN@Fe₃O₄–5 and hBN@Fe₃O₄–10 exhibited extensive Fe₃O₄ aggregation and surface coverage. The corresponding Fe and O elemental maps further confirmed the progressive increase in Fe₃O₄ deposition with increasing precursor feed amount. ICP analysis determined Fe₃O₄ contents of 4.2, 11.3, 29.0, and 44.7 wt% for hBN@Fe₃O₄–0.5, –2.5, –5, and –10, respectively (**Figure S6**). Their saturation magnetization values correspondingly increased to 3.2, 8.3, 20.3, and 33.3 emu g⁻¹, demonstrating that the magnetic response increased proportionally with the Fe₃O₄ content.

To evaluate the effect of Fe₃O₄ loading on thermal transport, epoxy composites containing 20 vol% raw hBN or hBN@Fe₃O₄ were prepared, and their through-plane thermal conductivities were measured before and after magnetic alignment (**Figure S7**). The non-aligned composites containing raw hBN, hBN@Fe₃O₄–0.5, hBN@Fe₃O₄–2.5, hBN@Fe₃O₄–5, and hBN@Fe₃O₄–10 exhibited thermal conductivities of 0.550, 0.552, 0.546, 0.429, and 0.358 W m⁻¹ K⁻¹, respectively. After magnetic alignment, the values for hBN@Fe₃O₄–0.5, –2.5, –5, and –10 increased to 1.008, 1.129, 0.908, and 0.674 W m⁻¹ K⁻¹, corresponding to enhancements of approximately 83, 107, 112, and 89%, respectively.

Although hBN@Fe₃O₄–0.5 maintained a high non-aligned thermal conductivity, its low saturation magnetization limited effective filler alignment. Conversely, the excessive Fe₃O₄ coverage and aggregation observed for hBN@Fe₃O₄–5 and –10 impeded thermal transport despite their stronger magnetic responses. Moreover, because the total filler loading was fixed at 20 vol%, increasing the Fe₃O₄ fraction reduced the effective hBN content. Consequently, hBN@Fe₃O₄–2.5 provided the optimal balance between magnetic responsiveness and preservation of the hBN-mediated heat-conduction pathway, producing the highest aligned through-plane thermal conductivity of 1.129 W m⁻¹ K⁻¹, corresponding to a 107% enhancement. Therefore, hBN@Fe₃O₄–2.5 was selected as the optimized formulation for the present study.

**Supporting Note S3. Torque–Viscosity Competition Governing Magnetic Alignment in Uncured Hybrid Composites**

Magnetic alignment of anisotropic fillers in uncured polymer composites is governed by a kinetic competition between field-induced rotational driving and viscous dissipation in the surrounding medium ^[5]^. For a magnetically responsive platelet, the magnetic torque tends to minimize the misorientation angle with respect to the applied field, whereas the viscous matrix resists rotational motion. As a result, the observed degree of alignment depends not only on the magnetic responsiveness of the filler itself, but also on whether the rheological state of the uncured composite allows sufficient rotational mobility during the available alignment time.

This framework is particularly relevant to the present Al₂O₃/Fe₃O₄-functionalized hexagonal boron nitride (hBN@Fe₃O₄)/epoxy system because increasing the fraction of hBN@Fe₃O₄ has two competing consequences. On the one hand, a higher flake fraction increases the number of magnetically addressable bridge fillers capable of participating in directional network formation. On the other hand, the same increase in high-aspect-ratio filler content also elevates viscosity and enhances filler–filler interactions, which increase rotational drag and suppress reorientation under a given magnetic field. Therefore, alignment efficiency is expected to show a non-monotonic dependence on hybrid filler composition, with the optimal condition emerging from a balance between magnetic programmability and rheological freedom.

In addition, the macroscopic anisotropy observed in SEM or FFT analysis does not necessarily scale linearly with the thermal importance of the aligned flake population. In sphere-dominant composites, a limited fraction of appropriately oriented bridge fillers may still have a pronounced influence on interparticle heat-flow continuity by selectively connecting neighboring spherical fillers across otherwise constricted junctions. Accordingly, the structural significance of magnetic alignment in the present system should be interpreted in terms of its effect on bridge-mediated connectivity, rather than only through the magnitude of the global anisotropy signal.

**Supporting Note S4. Non-Monotonic Optimization of Through-Plane Thermal Transport in Bridge-Filler Hybrids**

The composition dependence of through-plane thermal conductivity in the present Al₂O₃/hBN@Fe₃O₄ hybrid system should not be interpreted as a simple rule of “more anisotropic filler leads to higher thermal conductivity.” Instead, the observed optimum arises from the competition between two distinct effects. Increasing the hBN@Fe₃O₄ fraction increases the number of potential bridge fillers capable of connecting neighboring Al₂O₃ particles and reducing point-contact-dominated thermal resistance. At the same time, however, increasing the fraction of high-aspect-ratio flakes raises composite viscosity, enhances filler–filler interactions, and suppresses magnetic-field-induced reorientation ^[5]^. As a result, the thermally most effective state emerges at an intermediate bridge-filler fraction where connectivity gain and alignability are simultaneously optimized.

This framework also explains why the alignment-induced conductivity enhancement grows with total filler loading up to an intermediate-to-high loading regime but then weakens again at the highest loading. At low loading, bridge fillers are too spatially separated to strongly reorganize network continuity. At intermediate loading, neighboring spherical fillers are close enough that aligned flakes can effectively bridge constricted junctions and create more continuous through-plane pathways. At excessively high loading, however, the kinetic freedom required for field-induced reorientation is lost because of the large viscosity of the uncured composite ^[6]^. Therefore, the relationship between filler loading and performance is governed by a topology–mobility balance rather than by filler content alone. From a broader design perspective, the present results suggest that bridge-filler-enabled thermal transport should be optimized within a multidimensional window defined by filler geometry, bridge-filler fraction, magnetic responsiveness, and matrix rheology. This design principle is likely to be applicable beyond the specific Al₂O₃/hBN@Fe₃O₄ system investigated here.

**Supporting Note S5. Effect of hBN Lateral Size on Bridge-Network Formation and Through-Plane Thermal Transport**

To examine the effect of bridge-filler size on thermal-network formation, hBN flakes with lateral sizes of 5, 18, and 30 μm were functionalized with Fe₃O₄ using the same procedure. The 5 μm hBN corresponds to the filler used in the main study, whereas the 18 and 30 μm fillers were additionally examined for comparison. FE-SEM images confirmed successful Fe₃O₄ deposition on all three hBN surfaces (**Figure S10**). However, the 18 and 30 μm fillers exhibited greater Fe₃O₄ coverage and more localized particle aggregation than the 5 μm filler.

Each hBN@Fe₃O₄ filler was incorporated into epoxy at a fixed total filler loading of 40 vol% and an Al₂O₃:hBN@Fe₃O₄ volume ratio of 8:2. Cross-sectional BSE mode images of the composites before and after magnetic alignment showed clear through-plane reorientation of the hBN fillers for all three lateral sizes (**Figure S10**). Thus, the differences in thermal performance were evaluated in relation to the size-dependent network structures formed within the Al₂O₃-dominant composite.

As shown in **Figure S11**, the non-aligned composites containing 5, 18, and 30 μm hBN@Fe₃O₄ exhibited through-plane thermal conductivities of 1.211, 0.996, and 1.069 W m⁻¹ K⁻¹, respectively. After magnetic alignment, the corresponding values increased to 2.020, 1.302, and 1.401 W m⁻¹ K⁻¹, representing enhancements of 66.7, 30.7, and 31.1%, respectively. The 5 μm filler therefore produced both the highest thermal conductivity before and after alignment and the largest alignment-induced enhancement.

This behavior is attributed to the size-dependent connectivity of the hBN bridge network. At the same hBN volume fraction, the 5 μm platelets provide a larger number of individual bridge fillers and form a denser and more spatially uniform network between neighboring Al₂O₃ particles. In contrast, although the 18 and 30 μm platelets span longer distances, their lower number density limits the formation of uniformly distributed bridges, resulting in lower thermal conductivity and a smaller alignment-induced enhancement. In the present sphere-dominant hybrid system, where Al₂O₃ constitutes the major filler phase and hBN serves as a secondary bridge filler, increasing the lateral size of hBN is therefore not necessarily advantageous. Under the investigated filler composition and particle-size conditions, the 5 μm hBN@Fe₃O₄ filler provided the most effective bridge-network geometry and was identified as the optimal lateral size for through-plane thermal transport.

**Supporting Note S6. Relation between FEM, Effective-Medium Saturation, and Contact-Limited Heat Transport**

The finite-element method (FEM) analysis in the present study was intended to provide mechanistic rather than purely empirical insight into how bridge-filler alignment changes the topology of heat transport. In sphere-only systems, the main transport limitation does not arise solely from the intrinsic thermal conductivity of the filler phase, but from the constricted nature of inter-filler contacts. Because neighboring spherical particles form only limited real contact areas, heat must pass repeatedly through narrow junctions associated with strong local thermal resistance. Consequently, increasing filler conductivity beyond a moderate range does not proportionally increase the effective conductivity of the composite.

This interpretation is consistent with both the FEM results and the Lewis–Nielsen-type effective-medium prediction shown in **Figure S14**. Although the Lewis–Nielsen model does not explicitly resolve local heat-flux concentration, it captures the same macroscopic tendency toward diminishing returns at high filler conductivity in sphere-dominant particulate systems. The agreement between these two approaches supports the conclusion that saturation is a generic consequence of contact-limited transport rather than an artifact of a specific simulation geometry.

By contrast, when anisotropic flake fillers are introduced as aligned bridges, the network topology changes qualitatively. The bridge fillers reduce the number of severely constricted transport junctions and increase the continuity of the heat-flow pathway along the through-plane direction. Under these conditions, the composite can utilize the intrinsic conductivity of the filler phase more efficiently, so the effective conductivity continues to increase rather than saturating rapidly. In this sense, the role of the aligned bridge network is not simply additive; it is topological, because it changes the way thermal resistance is spatially distributed across the composite. The FEM model remains idealized and does not explicitly include every processing-dependent microstructural feature present in real composites. Nevertheless, its agreement with the experimental trends and with the effective-medium saturation behavior strongly supports the interpretation that thermal-bottleneck mitigation in the present system originates from bridge-enabled reorganization of inter-filler connectivity.

**Supporting Note S7. Interpretation of Dielectric Stability and IR Surface-Heating Behavior in the Optimized Hybrid Composites**

The dielectric behavior of the optimized Al₂O₃/hBN@Fe₃O₄ hybrid composite reflects a balance between filler-induced interfacial polarization and the retention of an electrically insulating matrix-dominant phase. Compared with neat epoxy, the Al₂O₃-only and aligned hybrid composites exhibit higher dielectric constants and loss tangents because the incorporation of ceramic fillers increases the number of heterogeneous filler–matrix interfaces and the associated interfacial polarization. Notably, the aligned hybrid composite exhibits lower dielectric constant and dielectric loss than the Al₂O₃-only composite over the investigated frequency range, indicating that the introduction and alignment of hBN@Fe₃O₄ bridge fillers do not impose an additional dielectric penalty relative to the conventional Al₂O₃-filled system. Although Fe₃O₄ is incorporated to provide magnetic responsiveness, the volume resistivity of the aligned hybrid composite remains on the order of ${10}^{15}\text{ }\Omega\cdot\mathrm{cm}$, confirming that the functionalized fillers remain electrically isolated and do not form a percolative electronic-transport pathway. Thus, the present hybrid architecture improves thermal transport through topological reorganization of phonon pathways rather than through formation of an electrically leaky conductive network.

The package-level IR thermal-imaging experiment should be interpreted as a macroscopic manifestation of through-plane heat-transfer efficiency rather than as a direct substitute for intrinsic thermal-conductivity measurement. Photographs of the chip mounted on the printed circuit board and cross-sectional views of the fabricated packages containing Al₂O₃-only, hybrid non-aligned, and hybrid aligned underfills are provided in **Figure S15**. During heating, a 100 °C external heater was applied to the chip for 150 s, and the temperature of the opposite PCB surface was monitored by IR thermal imaging. In this configuration, an underfill with more efficient through-plane heat transport transfers heat from the chip toward the PCB more rapidly, resulting in a faster increase in PCB surface temperature. After removal of the heater, the chip temperature was monitored during 180 s of natural cooling. A faster decrease in chip temperature indicates more efficient removal of residual heat through the underfill and PCB. Accordingly, the faster PCB heating and chip cooling observed for the hybrid aligned underfill are consistent with the independently measured through-plane thermal-conductivity enhancement and the bridge-enabled heat-flow continuity identified in the FEM analysis. The package-level IR measurement therefore provides an application-relevant complement to the LFA-based thermal-conductivity results.

**Supporting Methods S1. Image Analysis, Thermal Conductivity Calculation, and FEM Details**

Cross-sectional filler orientation before and after magnetic-field-induced alignment was analyzed from FE-SEM images using ImageJ software (National Institutes of Health, USA). For qualitative comparison of filler orientation, fast Fourier transform (FFT) patterns were generated from the SEM images using the same image-processing workflow for all samples. For the epoxy composite containing only hBN@Fe₃O₄ flakes, the alignment behavior was further evaluated by orientation-angle analysis. Individual flake domains were identified from the cross-sectional SEM images, and the angular distribution of the platelet orientation relative to the applied magnetic-field direction was plotted as a histogram before and after magnetic-field treatment.

The through-plane thermal conductivity ($\kappa$) of the composites was calculated from the measured thermal diffusivity ($\alpha$), density ($\rho$), and specific heat capacity ($C_{p}$) according to the relationship $\kappa=\alpha\rho C_{p}$. The through-plane thermal diffusivity was measured using a laser flash analyzer. The composite density was determined by the Archimedes method using an analytical balance equipped with a density kit (XPR205DR with Density Kit, METTLER TOLEDO, Switzerland). The specific heat capacity was measured using differential scanning calorimetry (DSC 214, NETZSCH, Germany).

Finite-element modeling (FEM) was carried out using COMSOL Multiphysics 6.2 (COMSOL Inc., USA), and the simulation geometries were generated using Blender (Blender Foundation, Netherlands). A cubic simulation domain of $30\text{ }\mu\text{m}\text{ }$was used to compare sphere-only, sphere + flake non-aligned, and sphere + flake aligned composite structures. Spherical fillers were modeled with a diameter of $5\text{ }\mu\text{m}$, and flake fillers were modeled with a lateral size of $5\text{ }\mu\text{m}$ and a thickness of $0.5\text{ }\mu\text{m}$. For the structure-comparison simulations, the total filler loading was fixed at 40 vol%, corresponding to the experimentally optimized condition. The lower and upper boundaries were set to 400 and 300 K, respectively, whereas the remaining boundaries were treated as adiabatic. The matrix thermal conductivity was set to 0.2 W m⁻¹ K⁻¹, the filler thermal conductivity was set to 50 W m⁻¹ K⁻¹, and the filler–matrix interfacial thermal resistance was fixed at $2.07\times{10}^{-8}\text{m}^{2}\text{K W}^{-1}$.

To examine the effect of intrinsic filler conductivity on composite heat transport, the filler thermal conductivity was additionally varied from 1 to 300 W m⁻¹ K⁻¹ over the filler-loading range of 0–50 vol%. The simulation results were further compared with experimentally measured thermal conductivities of commercial SiO₂/epoxy and Al₂O₃/epoxy composites to validate the modeling approach. A Lewis–Nielsen-type effective-medium calculation was additionally used to examine the saturation tendency of sphere-dominant particulate systems at high intrinsic filler conductivity. In this calculation, the matrix thermal conductivity was fixed at 0.2 W m⁻¹ K⁻¹, and the filler thermal conductivity was varied from 1 to 300 W m⁻¹ K⁻¹. This auxiliary analysis was used to support the interpretation that the limited thermal-conductivity enhancement in sphere-only composites originates from contact-limited transport rather than from insufficient intrinsic filler conductivity alone.

**Figure S1.** Morphology of the pristine thermally conductive fillers. FE-SEM images of the pristine fillers: (a) spherical Al₂O₃ particles and (b) hBN flakes.

**Figure S2.** Additional morphological and elemental characterization of hBN@Fe₃O₄. (a) Low- and high-magnification FE-SEM images of hBN@Fe₃O₄, showing nanoscale surface decoration while retaining the platelet morphology of hBN. (b) EDS elemental mapping images of B, N, Fe, and O for hBN@Fe₃O₄.

**Figure S3.** High-resolution XPS spectra of hBN@Fe₃O₄. (a) B 1s spectrum. (b) N 1s spectrum. (c) O 1s spectrum. (d) C 1s spectrum.

**Figure S4.** Thermal influence and quantitative analysis of PDA coating on hBN. (a) Thermogravimetric analysis (TGA) curves of pristine hBN, hBN@PDA, and PDA up to 700 °C. (b) Thermal conductivity of epoxy composites containing 20 vol% pristine hBN or hBN@PDA fillers.

 **Figure S5.** Morphological and elemental characterization of hBN@Fe₃O₄ prepared with different iron-precursor feed amounts. FE-SEM images and corresponding EDS elemental mapping images of B, N, Fe, and O for (a) hBN@Fe₃O₄–0.5, (b) hBN@Fe₃O₄–2.5, (c) hBN@Fe₃O₄–5, and (d) hBN@Fe₃O₄–10.

**Figure S6.** Magnetic properties and Fe₃O₄ contents of hBN@Fe₃O₄ prepared with different iron-precursor feed amounts. (a) Magnetic hysteresis loops of pristine hBN, hBN@Fe₃O₄–0.5, hBN@Fe₃O₄–2.5, hBN@Fe₃O₄–5, and hBN@Fe₃O₄–10. (b) ICP-derived Fe₃O₄ contents and corresponding saturation magnetization values of hBN@Fe₃O₄–0.5, hBN@Fe₃O₄–2.5, hBN@Fe₃O₄–5, and hBN@Fe₃O₄–10.

**Figure S7.** Fe₃O₄-loading-dependent thermal conductivity of hBN@Fe₃O₄/epoxy composites before and after magnetic alignment. Through-plane thermal conductivity of epoxy composites containing 20 vol% raw hBN, hBN@Fe₃O₄–0.5, hBN@Fe₃O₄–2.5, hBN@Fe₃O₄–5, or hBN@Fe₃O₄–10 fillers under non-aligned and magnetically aligned conditions.

**Figure S8.** Intrinsic magnetic alignability of hBN@Fe₃O₄ in the epoxy matrix. (a) Cross-sectional FE-SEM images of hBN@Fe₃O₄/epoxy composites containing 20 vol% hBN@Fe₃O₄ flakes before and after magnetic-field-induced alignment. (b) Orientation-angle distribution histograms obtained from ImageJ analysis, showing the increased preferential alignment of the flakes after magnetic-field treatment.

**Figure S9.** Orientation-angle distributions of hBN@Fe₃O₄ fillers in Al₂O₃/hBN@Fe₃O₄/epoxy composites before and after magnetic alignment. Orientation-angle distribution histograms derived from the cross-sectional FE-SEM images of non-aligned composites with Al₂O₃:hBN@Fe₃O₄ ratios of (a) 6:4 and (b) 8:2, and magnetically aligned composites with ratios of (c) 6:4 and (d) 8:2. The total filler loading was fixed at 40 vol%.

**Figure S10.** Morphology and size-dependent microstructures of hBN@Fe₃O₄ fillers and their hybrid epoxy composites. FE-SEM images of hBN@Fe₃O₄ fillers with lateral sizes of (a) 5 μm, (b) 18 μm, and (c) 30 μm. Cross-sectional FE-SEM images of Al₂O₃/hBN@Fe₃O₄/epoxy composites containing (d) 5 μm, (e) 18 μm, and (f) 30 μm hBN@Fe₃O₄ fillers before and after magnetic alignment. The total filler loading was fixed at 40 vol%, with an Al₂O₃:hBN@Fe₃O₄ volume ratio of 8:2.

**Figure S11.** Size-dependent thermal conductivity of Al₂O₃/hBN@Fe₃O₄/epoxy composites before and after magnetic alignment. Through-plane thermal conductivity of non-aligned and magnetically aligned composites containing hBN@Fe₃O₄ fillers with lateral sizes of 5, 18, and 30 μm. The total filler loading was fixed at 40 vol%, with an Al₂O₃:hBN@Fe₃O₄ volume ratio of 8:2.

**Figure S12.** FEM model geometries and temperature distributions at different filler loadings. FEM simulation models and corresponding temperature distributions of composites with total filler loadings of 10, 20, 30, 40, and 50 vol%: (a) sphere-only filler composites and (b) sphere + aligned flake hybrid filler composites.

**Figure S13.** Experimental validation of the FEM model. Comparison of experimentally measured and FEM-calculated thermal conductivity values for commercial SiO₂/epoxy and Al₂O₃/epoxy composites, validating the reliability of the simulation framework.

**Figure S14.** Effective-medium prediction of thermal-conductivity saturation behavior. Theoretical prediction of composite thermal conductivity based on the Lewis–Nielsen model, assuming a matrix thermal conductivity of 0.2 W m⁻¹ K⁻¹ and filler thermal conductivities of 1, 3, 5, 10, 30, 50, 100, and 300 W m⁻¹ K⁻¹.

**Figure S15.** Photographs of the fabricated semiconductor package and underfill configurations. (a) Top-view photograph of the chip mounted on the printed circuit board (PCB). (b) Cross-sectional side-view photographs of the bump-bonded semiconductor packages containing Al₂O₃, hybrid non-aligned, and hybrid aligned underfills, shown from top to bottom, respectively.

| Refs. | Matrix | Filler architecture/ surface functionalization | Total filler loading | Magnetic field | Thermal conductivity, aligned (W m⁻¹ K⁻¹) | Alignment-induced enhancement |
| --- | --- | --- | --- | --- | --- | --- |
| [7] | Waterborne poly-  urethane (WPU) | MnFe₂O₄-decorated BNNS | 30 wt% | 30–50 mT (0.03–0.05 T) | 8.5 (TP) | 35× vs neat WPU |
| [8] | PDMS | Fe₃O₄-decorated hBN nanosheets | 10 wt% | 1 T Halbach array | 3.584 (TP) | 1950% vs neat PDMS |
| [9] | TPU | Fe₃O₄ nanoparticles on BN nanosheets + TDI covalent modification | 30 wt% | 60 mT rotating magnetic field | 5.15 (IP) | 3012% increase vs neat TPU |
| [10] | PVA | PDA-assisted Fe₃O₄-functionalized BN nanosheets (Fe-PDA-BNNS) | 30 wt% | 0.3 T horizontal magnetic field | 5.237 (IP) | 20.5× vs neat PVA; 1.1× vs unaligned composite |
| [11] | Epoxy (DGEBA/  DDM) | Exfoliated BN nanoplatelets (BNNP) | 20 wt% | 0.4 T | 1.07 (TP) | 127.7% vs unaligned Fe₃O₄-BNNP composite; 72.6% vs raw BN composite |
| This work | Epoxy | Spherical Al₂O₃ + magnetically aligned hBN@Fe₃O₄ bridge fillers | 40 vol% total filler (32 vol% Al₂O₃ + 8 vol% hBN@Fe₃O₄) | 0.678 T | 2.020 (TP) | 66.7% vs unaligned hybrid composite |

TP = Through-plane thermal conductivity

IP = In-plane thermal conductivity

**Table S1.** Comparison of magnetically aligned BN-based thermally conductive polymer composites reported in the literature and comparison with the present work.

**References**

[1] Y. Yuan, W. Wu, H. Hu, et al., The Combination of Al₂O₃ and BN for Enhancing the Thermal Conductivity of PA12 Composites Prepared by Selective Laser Sintering, RSC Advances 2021, 11, 1984.

[2] A. G. M. Pornea, K. I. Choi, J. H. Jung, et al., Enhancement of Isotropic Heat Dissipation of Polymer Composites by Using Ternary Filler Systems Consisting of Boron Nitride Nanotubes, h-BN, and Al₂O₃, ACS Omega 2023, 8, 24454.

[3] Y. Liu, G. Zhu, F. Qiu, et al., Effect of h-BN Orientation on the Corrosion Resistance of Bioinspired Layered Phosphate Composite Coatings, ACS Applied Nano Materials 2024, 7, 20733.

[4] B. Zhai, L. Weng, X. Wang, et al., Fabrication and Thermal Conductivity Optimization of Epoxy Composites With Multidimensional Hybrid Fillers, Polymer Composites 2026, 47, 352.

[5] Y. Chen, Z. Gao, S. A. Hoo, et al., Sequential Dual Alignments Introduce Synergistic Effect on Hexagonal Boron Nitride Platelets for Superior Thermal Performance, Advanced Materials 2024, 36, 2314097.

[6] K. Kim, J. Kim, Vertical Filler Alignment of Boron Nitride/Epoxy Composite for Thermal Conductivity Enhancement via External Magnetic Field, International Journal of Thermal Sciences 2016, 100, 29.

[7] H. Jiang, J. Li, Y. Xie, et al., Design of Efficient Microstructured Path by Magnetic Orientation Boron Nitride Nanosheets/MnFe₂O₄ Enabling Waterborne Polyurethane with High Thermal Conductivity and Flame Retardancy, Journal of Materials Science & Technology 2025, 209, 207–218.

[8] V. Ghai, A. A. Mishra, E. Huang, et al., Halbach Array Induced Magnetic Field Alignment in Boron Nitride Nanocomposites, Advanced Science 2025, 12, 2408532.

[9] F. Yuan, W. Jiao, F. Yang, et al., Surface Modification and Magnetic Alignment of Hexagonal Boron Nitride Nanosheets for Highly Thermally Conductive Composites, RSC Advances 2017, 7, 43380–43389.

[10] H. Zhang, S. Xu, Magnetic Field-Assisted Alignment of Boron Nitride Nanosheets in Poly(vinyl alcohol) Composites with Enhanced In-Plane Thermal Conductivity, Journal of Polymer Research 2023, 30, 209.

[11] Y. Kim, J. Kim, Fabrication of Fe₃O₄-Coated Boron Nitride Nanoplatelets by Liquid-Phase Exfoliation for Thermally Enhanced Epoxy Composites via Magnetic Alignment, Composites Science and Technology 2020, 188, 107961.
